# Tumor suppression via inhibition of SWI/SNF complex-dependent NF-κB activation

**DOI:** 10.1038/s41598-017-11806-9

**Published:** 2017-09-18

**Authors:** Kazuyoshi Kobayashi, Hiroaki Hiramatsu, Shinya Nakamura, Kyousuke Kobayashi, Takeshi Haraguchi, Hideo Iba

**Affiliations:** 1 Division of Host-Parasite Interaction, Department of Microbiology and Immunology, The Institute of Medical Science, The University of Tokyo, Tokyo, 108-8639 Japan; 2 Division of RNA Therapy, Medical Mycology Research Center, Chiba University, Chiba, 260-8673 Japan

## Abstract

The transcription factor NF-κB is constitutively activated in many epithelial tumors but few NF-κB inhibitors are suitable for cancer therapy because of its broad biological effects. We previously reported that the d4-family proteins (DPF1, DPF2, DPF3a/b) function as adaptor proteins linking NF-κB with the SWI/SNF complex. Here, using epithelial tumor cell lines, A549 and HeLaS3, we demonstrate that exogenous expression of the highly-conserved N-terminal 84-amino acid region (designated “CT1”) of either DPF2 or DPF3a/b has stronger inhibitory effects on anchorage-independent growth than the single knockdown of any d4-family protein. This indicates that CT1 can function as an efficient dominant-negative mutant of the entire d4-family proteins. By *in situ* proximity ligation assay, CT1 was found to retain full adaptor function, indicating that the C-terminal region of d4-family proteins lacking in CT1 would include essential domains for SWI/SNF-dependent NF-κB activation. Microarray analysis revealed that CT1 suppresses only a portion of the NF-κB target genes, including representative SWI/SNF-dependent genes. Among these genes, *IL6* was shown to strongly contribute to anchorage-independent growth. Finally, exogenous CT1 expression efficiently suppressed tumor formation in a mouse xenograft model, suggesting that the d4-family proteins are promising cancer therapy targets.

## Introduction

Nuclear factor-kappa B (NF-κB) transcription factor (RelA, RelB, p50, and p52) regulates the expression of many genes involved in distinct biological processes, including development, differentiation, inflammation, viral propagation, and cell growth and survival^1,2^. In response to a range of stimuli, including pro-inflammatory cytokines, IκB kinase (IKK) is activated and phosphorylates IκB and marks it for polyubiquitination and degradation. The degradation of IκB, which forms a complex with NF-κB dimers, leads to exposure of the NF-κB nuclear localization signal, and translocation of the NF-κB dimer into the nucleus where it activates gene transcription^1,2^.

Dysregulated or constitutively activated NF-κB has been functionally linked to a wide variety of human diseases, including cancer and autoimmune and inflammatory disorders, caused by chronic inflammation^1–6^. Several low molecular weight^1–4^ and peptide inhibitors^7,8^ of NF-κB have been developed that target several key components of signaling pathways^5^ and ultimately inhibit the transactivation of NF-κB target genes. For example, a selective IKK inhibitor, NEMO binding domain (NBD) peptide efficiently blocks constitutive NF-κB activity and systemic administration of NBD peptide was shown to be successful in Phase 1 Clinical Trial in the suppression of Activated B Cell-like Diffuse Large B-Cell Lymphomas^9^.

However, because such key molecules, including cytokines and IκB kinases, reside at the intersection of several other important signaling pathways, these inhibitors might have unintended side effects. An attractive alternative target for inhibition therefore would be the most downstream step in the NF-κB signaling pathway i.e. the transactivation step. But, when such inhibitors are systematically administered as cancer therapeutics, there is the possibility that they may suppress immune responses such as the tumor-eliminating functions of immune cells^1,2^. Given such a possible immunological deterioration in cancer patients, suppression of all NF-κB target genes would not necessarily be successful.

The SWI/SNF complex is a representative chromatin-remodeling factor involved in epigenetic regulation in humans^10,11^. This complex has a single molecule of either BRG1 or Brm as the catalytic subunit, but not both. A lack of endogenous Brm expression has been shown to have broad biological effects including the loss of anchorage-independent growth^12^. Individual members of the d4-family proteins, DPF1 (NEUD4/BAF45B), DPF2 (UBID4/REQ/BAF45D), DPF3a (CERD4/BAF45C), and DPF3b (a splicing variant of DPF3a) interact with the SWI/SNF complex^13–15^. We have previously shown that DPF2 functions as an efficient adaptor protein between the SWI/SNF complex and the RelB/p52 dimer that functions at the most downstream step of the non-canonical NF-κB pathway^16^. We further reported that high expression levels of any of the four d4-family proteins can potentiate the transactivating activity of the typical NF-κB dimer RelA/p50, which is responsible for the canonical NF-κB pathway, as well as RelB/p52^17^. In addition, we demonstrated that, of these four proteins, DPF3a and DPF3b are the most effective cofactors for RelA/p50 activation in 293FT cells.

The induction of genes by several stimuli in immune cells can be either dependent or independent on the SWI/SNF complex^16,18,19^. In the present study, we investigated the development of inhibitors of the SWI/SNF complex-dependent activation of NF-κB target genes to ultimately test whether such inhibitors can suppress epithelial tumors. We first found that in several epithelial tumor cell lines, the knockdown of any of these proteins reduced anchorage-independent growth to some extent, whereas these cells expressed *DPF1*, *DPF2*, *DPF3a*, and *DPF3b* mRNA at various levels. We found that a peptide composed of the 84 N-terminal amino acids shared among the d4-family (named “CT1”) retains full adaptor activity linking the SWI/SNF complex and NF-κB but lacks NF-κB transactivating activity. Hence, CT1 can function as a strong dominant-negative mutant by competing with the adaptor function of all d4-family proteins. CT1 can also specifically suppress the induction of a small subset of TNF-α inducible NF-κB target genes, and also anchorage-independent growth, much more effectively than any single knockdown of a d4-family protein. Finally, we show that CT1 suppresses tumorigenicity in a mouse xenograft model.

## Results

### A single knockdown of any d4-family member moderately suppresses the anchorage-independent growth of epithelial tumor cell lines

We first tested the impact of the knockdown of each d4-family member on anchorage-independent growth using human cancer cell lines originating from non-small cell lung carcinoma (A549) (lacking BRG1 expression) and cervical tumor (HeLaS3) (competent for both BRG1 and Brm expression) using a series of short-hairpin (sh) RNAs^12,17,20^. All of these shRNAs expressed from lentivirus vectors were previously shown to suppress the corresponding mRNAs at least to 40% of those in cells carrying empty vector. The suppression of anchorage-independent growth by these knockdowns of d4-family members in these two cell lines is basically moderate (20–80%) compared to knockdown of Brm or BRG1 (15–40%) in HeLaS3 and knockdown of Brm in A549 (15–20%) (Supplementary Fig. S1), whereas DPF2 knockdown in A549 showed somehow profound effects. It should be pointed out that each knockdown of d4-family genes only marginally affected cellular growth in monolayer cultures (Supplementary Fig. S2). These results are consistent with our previous observations in H1299 and Panc-1 cells expressing shDPF2^16^ and further suggest that a single knockdown of any d4-family protein can partially block the link between the SWI/SNF complex and NF-κB dimers. However, the remaining d4-family members are still expressed in the single-knockdown cells and the gene expression levels of the d4-family members vary among epithelial tumor cell lines (Supplementary Fig. S3). Therefore, simultaneous suppression of all d4-family proteins would be required for developing new NF-κB inhibitors, which effects on all tumor cell types.

### Truncated d4-family protein functions as a dominant-negative mutant

**Figure 1:**
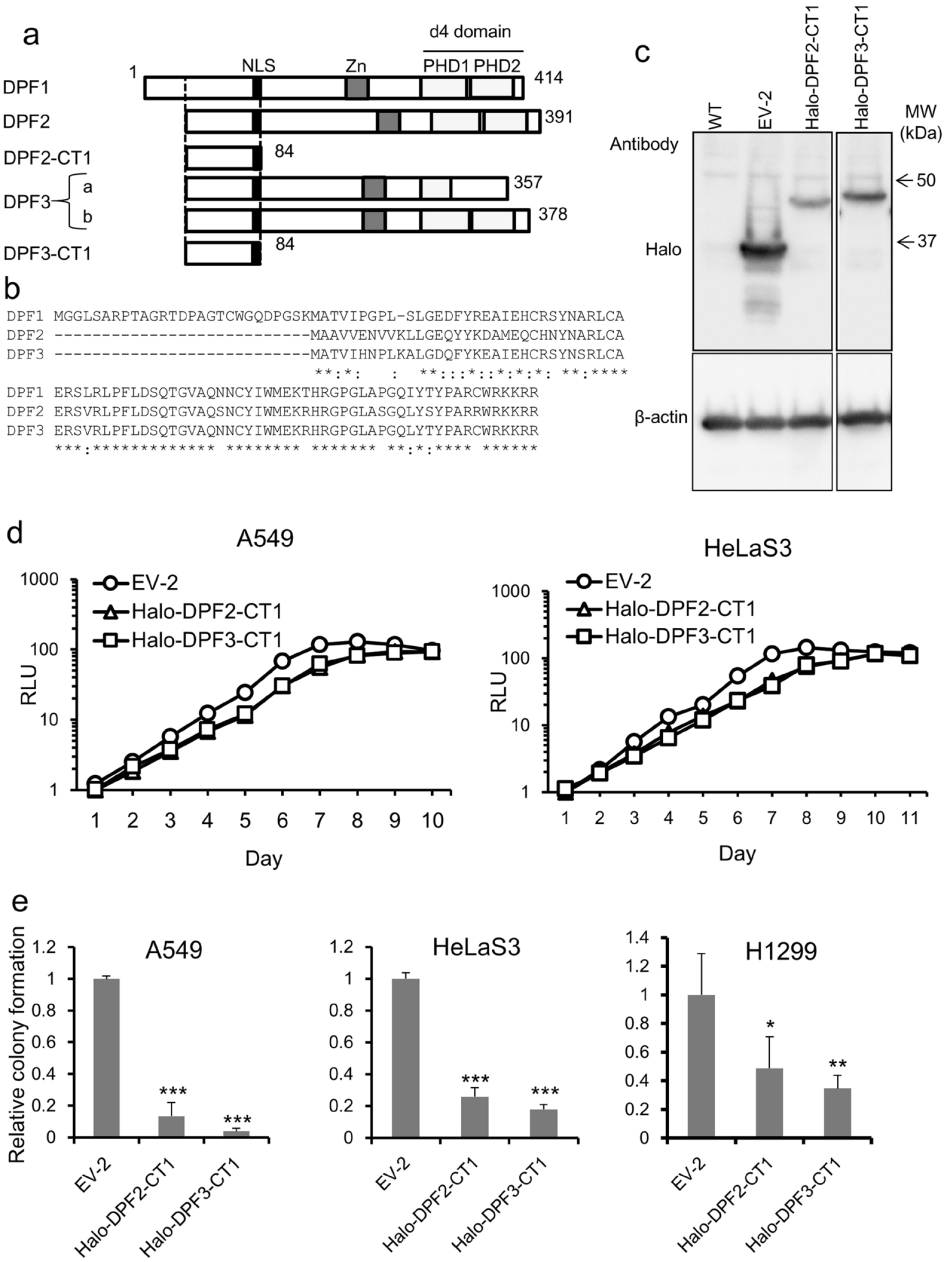
The structure and properties of d4-family proteins and their truncation mutant, CT1. (**a**) Schematic representation of the structure of the d4-family members and the CT1 truncation mutant. NLS, nuclear localization signal (black). Zn, C2H2-type zinc finger motif (grey). PHD1 and PHD2, the tandem PHDs (plant homeodomains) (light grey). The box shown by the broken line indicates the N-terminal region conserved among the d4-family proteins. (**b**) Amino acid alignment of the N-terminal regions of DPF1, DPF2, and DPF3. DPF1 has an additional N-terminal sequence composed of 27 amino acids. The asterisks indicate identical residues. Colons indicate positions where the amino acid exchange is highly conservative. (**c**) Halo tag protein expression in monolayer cultures. A549 cells were transduced with lentivirus vector containing Halo-DPF2-CT1 at an MOI of 3.0. After puromycin selection, total proteins were prepared and protein expression was analyzed by western blotting using anti-Halo antibody. β-actin was used as the internal control. (**d**,**e**) Effects of exogenous expression of Halo-DPF2-CT1 and Halo-DPF3-CT1 on the growth of monolayer cultures (**d**) and soft agar cultures (**e**). Using a parallel culture as described in (**c**), cells were seeded into 96-well plates and growth was then monitored by a CellTiter-Glo luminescent cell viability assay every 24 h. Cells in a parallel culture were suspended in soft agar in 60-mm plates and colonies (more than 100 mm in diameter) were counted 18–25 days after seeding. Colony numbers formed by cells transduced with EV-2 were assigned a value of 1.0. Colony numbers for H1299 cells were determined in the same procedure. Similar results were obtained from at least two independent experiments. The data present means ± SD (*n* = 3). *P < 0.05, **P < 0.01, ***P < 0.001.

The d4-family proteins harbor a conserved N-terminal domain, a central C2H2-type zinc finger motif, and C-terminal paired plant homeobox domain (PHD) fingers that have been suggested to mediate their interactions with other proteins, including modified histones^13,21,22^ (Fig. 1a). We previously showed that a GST-DPF2 fusion protein can directly bind multiple SWI/SNF subunits, Brm, BRG1, Ini1, and BAF60A and p52 (an NF-κB subunit) *in vitro* ^16^. We had also constructed a series of truncation mutants of DPF2 and tested their ability to bind these SWI/SNF subunits or p52 proteins. These previous observations indicated that only a limited portion of the N-terminal region of DPF2 (from residues 1 to 84; hereafter designated the “CT1” peptide; Fig. 1a) is sufficient for binding. When the amino acid sequences of all d4-family proteins were aligned and compared, we found that the CT1 region was highly conserved among all the member proteins (DPF3a and DPF3b have the same N-terminal sequence) (Fig. 1b). The 8 C-terminal amino acids of CT1 comprise a representative nuclear localization signal that would facilitate the transport of CT1 to the cell nucleus. Thus, we hypothesized that the CT1 region includes the essential domain for the adaptor function linking the SWI/SNF complex and NF-κB dimer and could function as a putative dominant-negative mutant that inhibits the entire suite of d4-family proteins when expressed at high levels.

### The CT1 peptide inhibits the anchorage-independent growth of epithelial tumor cells

We fused the CT1 peptide of DPF2 or DPF3 to a Halo tag (designated Halo-DPF2-CT1 or Halo-DPF3-CT1, respectively) and could stably express them (Fig. 1c), consistent with the previous report that the Halo tag often stabilizes short peptides^23^. The expression of these fusion proteins strongly inhibited colony formation in soft agar: colony numbers were drastically reduced when compared with cells transduced with an empty vector (EV-2), whereas they only slightly reduced the growth rate of monolayer cultures (Fig. 1d and e). Such reduction in anchorage-independent growth was also observed in another non-small cell lung carcinoma cell line, H1299 (Fig. 1e). Overall, these results suggested that CT1 functions as a dominant-negative mutant for all of the wild-type d4-family proteins and also that SWI/SNF-dependent NF-κB activity would have more profound effects on colony forming activity in soft agar than on growth in monolayer culture. Notably, DPF3-CT1 suppressed anchorage-independent growth more efficiently than DPF2-CT1 in both HeLaS3 and A549 cells. This might reflect the fact that Halo-DPF3-CT1 was expressed at higher levels than Halo-DPF2-CT1 from the same vector (Fig. 1c).

### A specific subset of NF-κB target genes requires the SWI/SNF complex for gene expression

We previously found that the shRNA knockdown of each d4-family protein, as well as Brm and BRG1, suppressed the luciferase activity of an NF-κB reporter integrated into the cellular chromosome^17^. In addition, several groups including our own have reported that only a specific subset of endogenous genes targeted by stimuli-activated transcription factors requires chromatin remodeling by the SWI/SNF complex^16,18,19,24–26^. To obtain deeper insights into the molecular mechanisms underlying CT1 function, we first examined the SWI/SNF dependency of representative NF-κB target genes. We chose four genes—*IL6* ^27,28^, *IL8* ^29^, *TNF* ^30,31^, and *ICAM1* ^32^—because promoter analysis of several tumor cell lines showed that they are induced by TNF-α, a representative inducer of NF-κB activation, through NF-κB binding site(s) present in their promoters. In A549 cells (deficient in BRG1 expression), a knockdown of Brm drastically reduced the high level of induction of both *IL6* and *IL8* by TNF-α compared with *TNF* and *ICAM1* induction (Supplementary Fig. S4a). This clear difference in the Brm dependency of these four genes was detectable even at the basal expression level (Supplementary Fig. S4d). In HeLaS3 cells, a knockdown of either Brm or BRG1 moderately reduced the induction of both *IL6* and *IL8* by TNF-α (Supplementary Fig. S4b). But when HeLaS3 cells in which both Brm and BRG1 were simultaneously knocked down, induction of *IL6* and *IL8* were drastically reduced, whereas induction of *TNF* and *ICAM1* were not so much affected (Supplementary Fig. S4c). From these results, we think both Brm-type and BRG1-type SWI/SNF complex similarly contribute to NF-κB transactivation in the *IL6* and *IL8* promoters.

When SW13 cells, which do not express either Brm or BRG1, were stably introduced with exogenous Brm or BRG1, the basal expression of the *IL6* and *IL8* genes dramatically increased by more than 37-fold compared with SW13 cells transduced with an empty vector (Supplementary Fig. S4c). Compared with these two genes, the basal expression of the *TNF* and *ICAM1* genes were not so much affected by exogenous Brm or BRG1 expression. Overall, these loss-of-function and gain-of-function experiments indicated that NF-κB target genes can be roughly categorized into two subtypes according to their SWI/SNF dependency: in these epithelial tumor cell lines, the *IL6* and *IL8* genes require the SWI/SNF complex for their activation through NF-κB whereas *TNF* and *ICAM1* do not.

### CT1 inhibits the induction of a specific subset of NF-κB target genes

**Figure 2:**
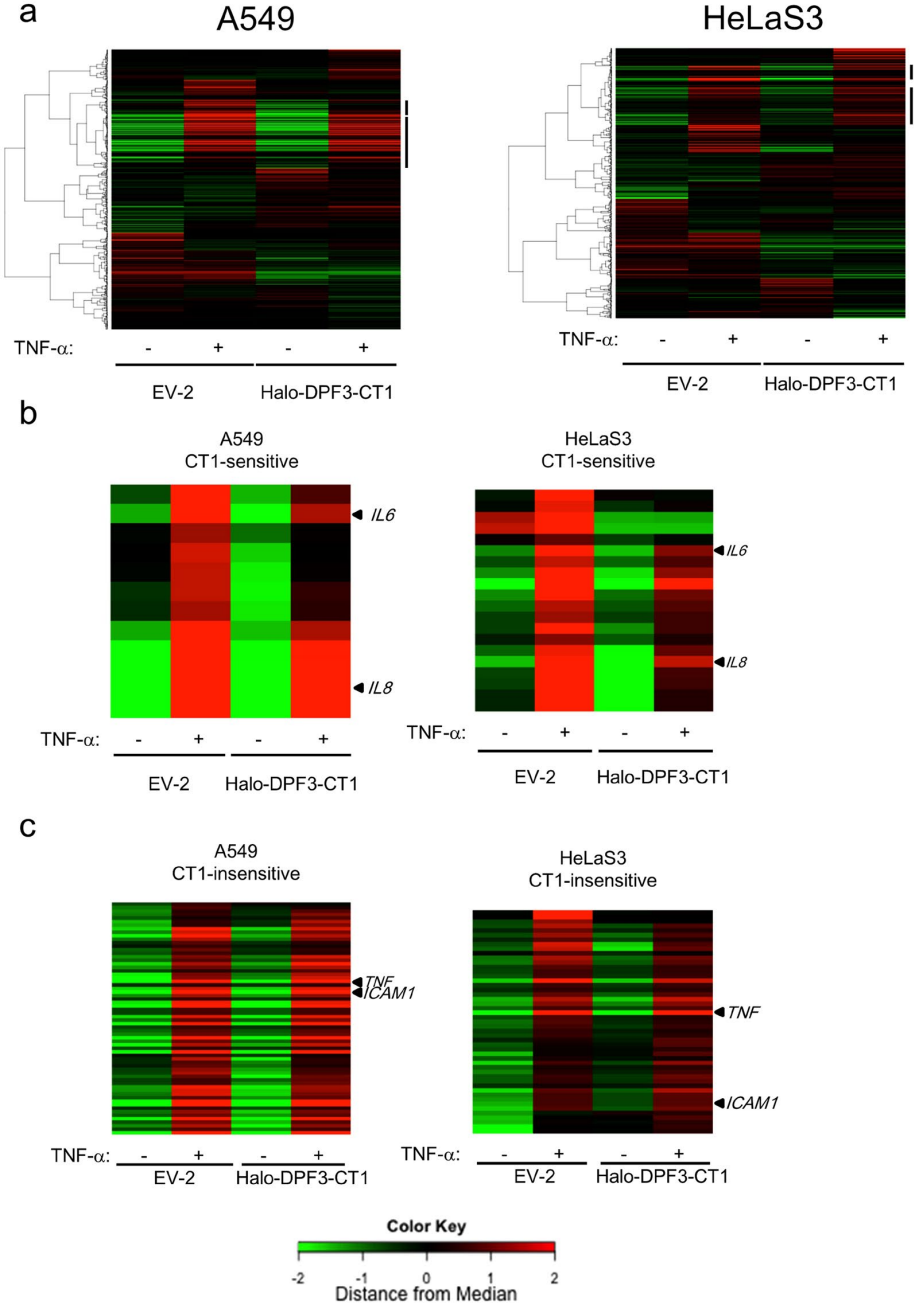
Microarray analysis of NF-κB target genes induced by TNF-α in A549 and HeLaS3 cells. (**a**) Heat map generated by microarray analysis of reported NF-κB target genes. Cells transduced with Halo-DPF3-CT1–expressing retrovirus vector or empty vector (EV-2) were grown in the absence (TNF-α: −) or presence (TNF-α: +) of TNF-α (10 µg/ml) for 1 h. Total RNA was isolated and analyzed by microarray as described in the Materials and Methods. Quantile normalized expression data were calculated using R package. All reported NF-κB target genes (Boston University; http://www.bu.edu/nf-kb/) were clustered using four RNA samples (EV-1 ± TNF-α and Halo-DPF3-CT1 ± TNF-α). Bars on the right indicate the gene cluster regions including TNF-α inducible genes in these cells. (**b**,**c**) Heat map of TNF-α–induced NF-κB target genes that were either CT1-sensitive or CT1-insensitive. From the list of NF-κB target genes, we selected TNF-α inducible genes in A549 or HeLaS3 cells using the following criteria: expression ratio of TNF-α–treated EV-1 transduced cells to untreated EV-1 transduced cells >1.5 and a Z-score > 2. From these TNF-α inducible genes in either A549 or HeLaS3 cells (a total of 71 genes), CT1-sensitive genes (**b**) were further selected by the following criteria: ratio of CT1-expressing cells to EV-2 transduced cells <0.66 and a Z-score < −2. The other genes were classified as CT1-insensitive (**c**).

Gene promoters containing NF-κB binding sites show wide variations in inducibility even after treatment with TNF-α. The induction would be affected by several factors, including the chromatin contents of the promoters in the cell type used and several other transcription factors that would be secondarily induced by NF-κB after the TNF-α treatment. We performed microarray analysis to determine the CT1 sensitivity of the NF-κB target genes induced by TNF-α treatment in these two cell lines (Fig. 2a). We tested the NF-κB target genes that had been confirmed previously by promoter analysis (https://www.bu.edu/nf-kb/; 594 probes for NF-κB target genes). The results indicated that 54 genes in A549 cells and 50 genes in HeLaS3 cells were induced more than 1.5-fold by a 1 hour treatment with TNF-α (Fig. 2b and c and Supplementary Table S1). The 71 genes induced by TNF-α in either A549 or HeLaS3 cells included 17 cytokines. Among these cytokine genes, 6 were commonly suppressed to less than 66% in CT1-expressing cells when compared with cells transduced with EV-2 (Fig. 2b and c). The *IL6* and *IL8* genes, the induction of which were shown to require the SWI/SNF complex in our earlier qRT-PCR analysis (Supplementary Fig. S4), were included among the CT1-sensitive genes as expected.

**Figure 3:**
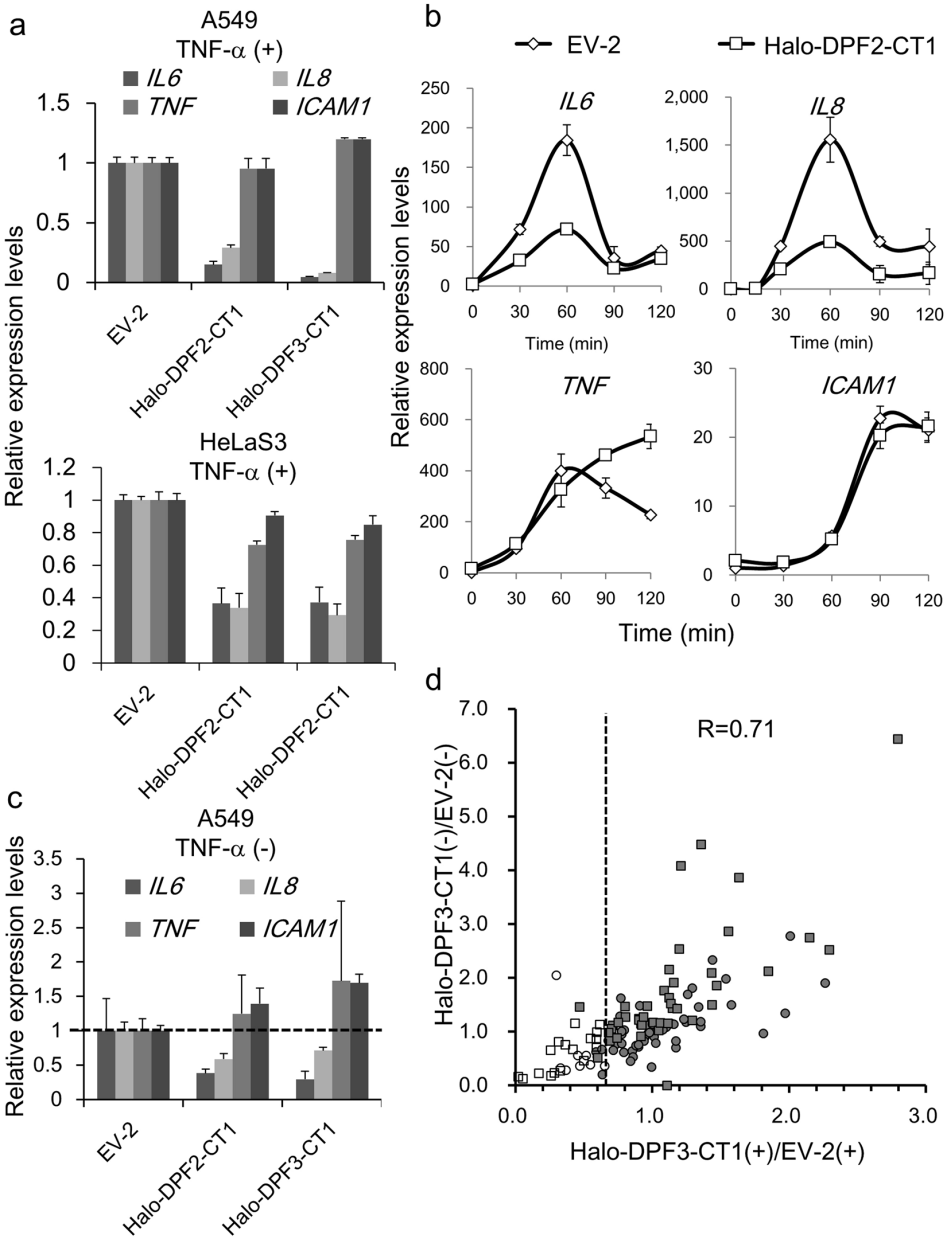
Suppression of TNF-α inducible NF-κB target genes by CT1. (**a**,**b**) A549 or HeLaS3 cells were transduced with Halo-DPF3-CT1 expressing retrovirus vector or empty vector (EV-2). The cells were then stimulated with TNF-α for 1 h (**a**) or for 30, 60, 90, and 120 min (**b**) and total RNA was prepared. The mRNA levels of *IL6*, *IL8*, *TNF*, and *ICAM1* were determined by qRT-PCR. The ratios of these mRNA levels to those of A549 (or HeLaS3) cells that were transduced with EV-2 (control) are shown in (**a**). In (**b**), RNA levels before the TNF-α treatment were taken as 1.0. (**c**) RNA was extracted from parallel cultures that were maintained in the absence of TNF-α treatment, and the mRNA levels of these four genes were determined and presented as in (**a**). (**d**) Scatter diagram comparing the effect of CT1 suppression on the basal expression (CT1–/EV-2−) and TNF-α–induced expression (CT1+/EV-2+) of all NF-κB target genes that were induced by TNF-α in either A549 or HeLaS3 cells. The NF-κB target genes listed in Supplementary Table S1 were plotted (ratio of EV-2(+) to EV-2(−) > 1.5, Z-score > 2.0). White boxes: CT1-sensitive genes in HeLaS3, Grey boxes: CT1-insensitive genes in HeLaS3. White circles: CT1-sensitive genes in A549, Grey circles: CT1-insensitive genes in A549. (**a**–**c**), The data present means ± SD (*n* = 3).

In contrast, *ICAM1* and *TNF*, which we has already categorized as SWI/SNF-independent NF-κB target genes, were detected among the 27 genes that were TNF-α inducible but not sensitive to CT1 suppression in either A549 and HeLaS3 cells. When the same RNA samples were also analyzed by qRT-PCR, the results more clearly confirmed the microarray findings of the CT1 sensitivity of *IL6* and *IL8* and CT1 insensitivity of *TNF* and *ICAM1* (Fig. 3a). Importantly, these 27 genes contained transcription factors including NF-κB genes (*NFKB1*, *NFKB2*, and *RELB*) and their regulators (*NFKBIA* and *NFKBIB*) that function in the inflammatory response of immune cells, suggesting a preference of these genes for SWI/SNF independence. When the presence or absence of CpG islands in the promoter regions was screened using the UCSC Genome Browser database, we found considerably enrichment in the promoter regions of CT1-insensitive genes (33/58) compared with CT1-sensitive genes (4/13) (Table 1). This suggested that genes with a broad-based expression profile such as housekeeping genes^33^ are SWI/SNF-independent (Tables 1 and S1).

**Table 1:** CT1- sensitivity of NF-κB target genes.

CT1-Sensitivity	Gene	CpG	Cytokine activity	Transcriptional factor
CT1-sensitive	*CCL20*	−	+	−
	*CXCL1*	+	+	−
	*DUSP5*	+	−	−
	*IL6*	−	+	−
	*IL8* (*CXCL8*)	−	+	−
	*INHBA*	−	+	−
CT1-insensitive	*BCL3*	+	−	−
	*CCL2*	−	+	−
	*CD83*	+	−	−
	*EBI3*	−	+	−
	*ICAM1*	+	−	−
	*IER2*	+	−	+
	*IL1A*	−	+	−
	*IL23A*	−	+	−
	*IRF1*	+	−	+
	*JUNB*	+	−	+
	*KDM6B*	+	−	−
	*NFKB1*	+	−	+
	*NFKB2*	+	−	+
	*NFKBIA*	+	−	+
	*NFKBIE*	+	−	+
	*NR4A2*	+	−	+
	*NUAK2*	+	−	−
	*PIM1*	+	−	−
	*PLK3*	+	−	−
	*RELB*	+	−	+
	*SAT1*	+	−	−
	*SDC4*	+	−	−
	*SOD2*	+	−	−
	*STAT5A*	+	−	+
	*TICAM1*	+	−	−
	*TNF*	−	+	−

Summary slide of CT1-sensitive and CT1-insensitive genes found in both A549 and HeLaS3 cells that were extracted from Supplementary Table S1. “Cytokine activity” and “Transcription Factor” genes were categorized by Gene Ontology analysis using David (https://david.ncifcrf.gov/). The CpG content of each gene promoter was evaluated with the UCSC genome Brower and the promoters whose CpG island size was larger than 300 were considered to be CpG (+).

Detailed kinetic analysis of *IL6*, *IL8*, *TNF* and *ICAM1* by qRT-PCR confirmed that they are all primary response genes in this cellular context and, furthermore, that CT1 suppressed *IL6* and *IL8* without affecting the induction profiles, at least within 100 min after TNF-α stimulation (Fig. 3b). When A549 and HeLaS3 cells were maintained in normal medium, these four genes were expressed at basal levels which were still detectable by qRT-PCR in A549 cells. Importantly, the basal expression of both *IL6* and *IL8* was sensitive to CT1 expression, but that of the *TNF* and *ICAM1* genes was largely CT1-insensitive (Fig. 3c), indicating that the categorization of these four genes by CT1 sensitivity was unchanged between basal expression conditions and after TNF-α stimulation.

Using microarray data, we next plotted a scatter diagram of the basal and TNF-α–induced expression levels of NF-κB target genes in A549 and HeLaS3 cells (Fig. 3d). The results indicated that the CT1 sensitivity of the basal expression of each NF-κB target gene correlates well to that of the TNF-α induced expression level (R = 0.71).

HA-tagged CT1 also functions as an efficient dominant-negative mutant and retains its adaptor function linking the SWI/SNF complex and NF-κB.

**Figure 4:**
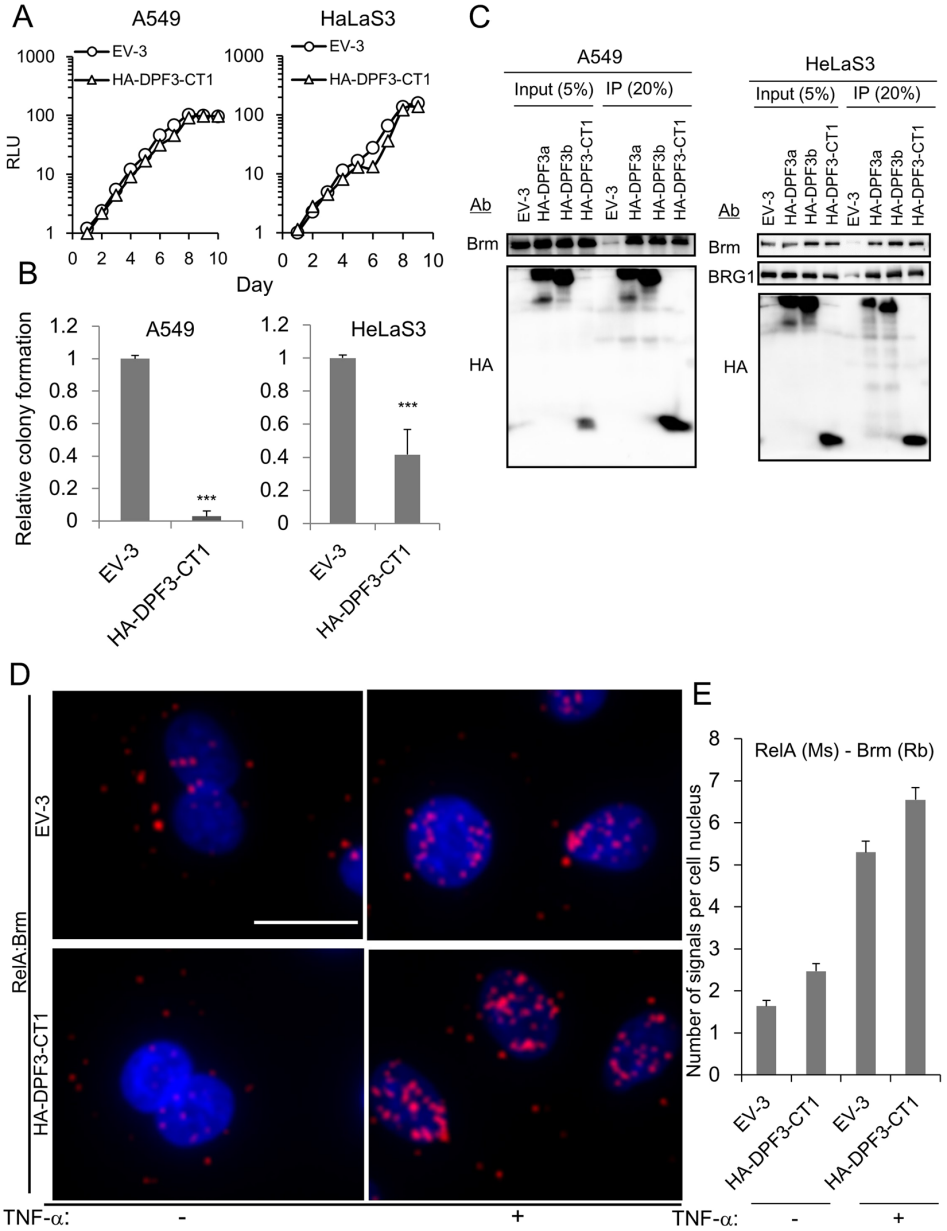
Properties of HA-DPF3-CT1 in cellular growth and its binding activity to the SWI/SNF complex. (**A**,**B**) Effects of exogenous expression of HA-DPF3-CT1 on the cell growth of monolayer cultures (**A**) and soft agar cultures (**B**) examined by the same procedure described for Fig. 1d and e. (**C**) Coimmunoprecipitation of catalytic subunit(s) of the SWI/SNF complex with HA-DPF3-CT1, HA-DPF3a, and HA-DPF3b. Cell nuclei were prepared from cells expressing HA-DPF3a, HA-DPF3b, or HA-DPF3-CT1, immunoprecipitated with anti-HA antibody, and separated by polyacrylamide gel electrophoresis. Brm and BRG1 were detected by western blotting. (**D**) A549 cells transduced with EV-3 or HA-DPF3-CT1 were grown in the absence (TNF-α: −) or presence of TNF-α for 20 min (TNF-α: +) and then fixed. *In situ* PLA was performed using proximity probes against Brm and RelA to detect close localization between these proteins. The cells were counterstained with DAPI (nuclei). Bar, 20 μm. (**e**) Red dot *in situ* hybridization signals in cellular nuclei were counted using 50 cells each. Error bars represent the means ± SEM. Similar results were obtained from at least two independent experiments. **A**, **B**, **D**, Similar results were obtained from at least two independent experiments. ***P < 0.001.

Considering the future development of peptide NF-κB inhibitors, the 33 kDa Halo tag would be too bulky. Our preliminary experiments indicated that HA-CT1 showed a relative stability that was comparable to Halo-CT1 in cells and a similar but slightly reduced dominant-negative ability. HA-CT1 reduced anchorage-independent growth without affecting the growth of monolayer cultures (Fig. 4A and B). To test whether HA-CT1 could bind Brm or BRG1 in cells, total cell lysates were immunoprecipitated with anti-HA antibody. Both Brm and BRG1 were coimmunoprecipitated in HeLaS3 cells at a similar efficiency as full-length HA-DPF3a or HA-DPF3b (Fig. 4C). In A549 cells, which lack BRG1, Brm was successfully coimmunoprecipitated with HA-CT1.

A large protein complex including the SWI/SNF complex and NF-κB has not been detected previously by coimmunoprecipitation, including our own previous report^16,17,34^; Immunoprecipitates generated with antibodies against SWI/SNF subunits do not contain NF-κB dimers and *vise versa*. This might reflect the instability of this large protein complex in cells or an equilibrium between association and dissociation of NF-κB dimers with complexes. To monitor the proximal localization of the SWI/SNF complex and NF-κB, we used an *in situ* proximity ligation assay (PLA)^35,36^. After fixing the cells, anti-Brm and anti-RelA primary antibodies were added and the localization of the resulting signals was assessed by PLA. When EV-3 transduced A549 cells were treated with TNF-α for 30 min, strong signals were detectable in the nuclei, indicating a close proximity of NF-κB to the SWI/SNF complex after its nuclear translocation (Figs 4D and E and S5). These observations suggested that a large complex including SWI/SNF complex and NF-κB dimer is formed via the endogenous d4-family proteins. Importantly, when HA-DPF3-CT1 was exogenously expressed in A549 cells, the signals in cellular nuclei were slightly enhanced (Fig. 4D and E), indicating that CT1 retains adaptor function and links the SWI/SNF complex and NF-κB. These *in vivo* results are consistent with our previous observations that CT1 can bind multiple SWI/SNF complex subunits (Brm/BRG1, BAF60A, and Ini1) and NF-κB dimers (p50 and RelA) *in vitro* ^16^.

To map the region required for the dominant-negative function of CT1, several truncation or deletion mutants of CT1 were constructed (Supplementary Table S2). Whereas the Δ40–76 and Δ53–76 CT1 mutants no longer suppressed *IL6* gene induction by TNF-α or anchorage-independent growth, the Δ1–8 product retained these dominant-negative activities to some extent. We cannot conclude that the 39 N-terminal residues of CT1 are dispensable for its dominant-negative function because both the Δ1–39 and Δ1–32 mutants were unstable and undetectable when expressed in A549 cells using virus vectors (Supplementary Table S2).

### IL-6 is intensely involved in anchorage-independent growth activity

**Figure 5:**
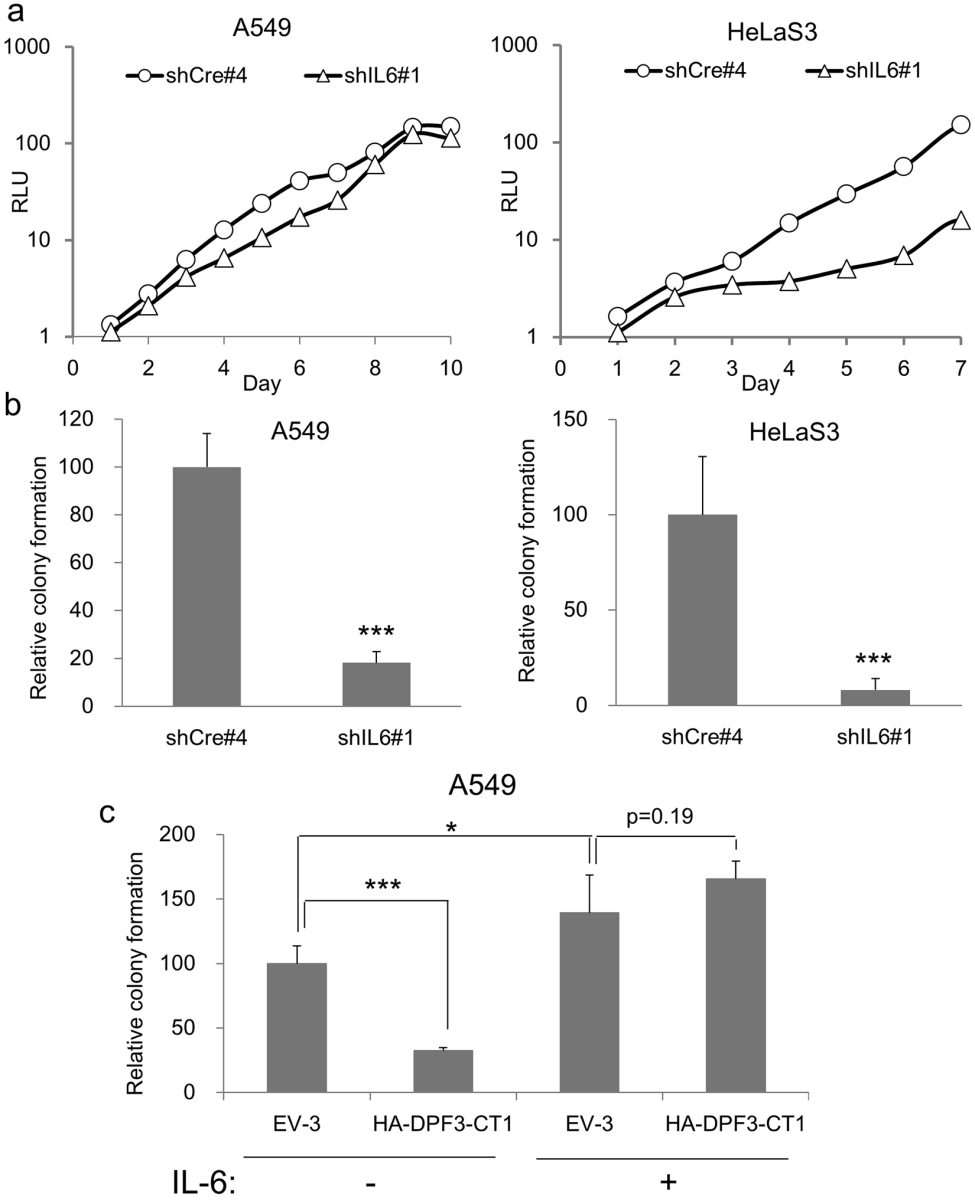
Effects of *IL6* knockdown or the addition of IL-6 protein on cellular growth. (**a**,**b**) Effects of shIL6 expression on the growth of monolayer cultures (**a**) and soft agar cultures (**b**) examined by the same procedure described for Fig. 1d and e. A549 or HeLaS3 cells were transduced with shRNA expressing vectors. The knockdown efficiency of shIL6#1 was calculated as 0.28 ± 0.014 in MDA-MB-231 cells. Colony numbers formed by cells transduced with shCre#4 (negative control) were assigned a value of 1.0. (**c**) The suppression of anchorage-independent growth by the exogenous expression of HA-DPF3-CT1 can be rescued by IL-6. A549 cells transduced with HA-DPF3-CT1 were kept in soft agar in the absence or presence of IL-6 (10 ng/ml). Colony numbers formed by cells transduced with EV-3 were assigned a value of 1.0. (**a**,**b**,**c**) The data present means ± SD (*n* = 3). *P < 0.05, ***P < 0.001.

Given that CT1 strongly suppressed the anchorage-independent growth of HeLaS3 and A549 cells, we hypothesized that some of the CT1-sensitive genes in these cells would be involved in the transformation pathways that enabled this growth. Among the candidate genes on this list (Table 1), we focused on the IL-6 cytokine as it is reported to have multiple functions including the maintenance of cancer stem cells and inhibition of apoptosis^37–40^. By introducing shIL6, we found that A549 cells had reduced colony forming activity in soft agar but grew normally in monolayer culture (Fig. 5a and b). In HeLaS3 cells however, the introduction of shIL6 reduced both colony forming activity in soft agar as well as the growth rate in monolayer culture (Fig. 5a and b). These results indicated that basal level of *IL6* gene expression is important for anchorage-independent growth in HeLaS3 and A549 cells, but the underlying molecular mechanisms may differ between these two cell lines. We next tested whether the suppression of colony formation by CT1 could be rescued by introducing exogenous IL-6 protein into the soft agar. Indeed, the presence of 10 ng/ml of IL-6 fully rescued the colony forming activity of HA-CT1 expressing cells to the same level as EV-3 expressing A549 cells (Fig. 5c). Together with the previous report that A549 failed to colonies in soft agar containing anti-IL-6 antibodies^37^, these results would indicated that *IL6* is one of the critical CT1-target genes that supports oncogenic potential.

### CT1 strongly suppresses tumorigenicity in a mouse xenograft model

**Figure 6:**
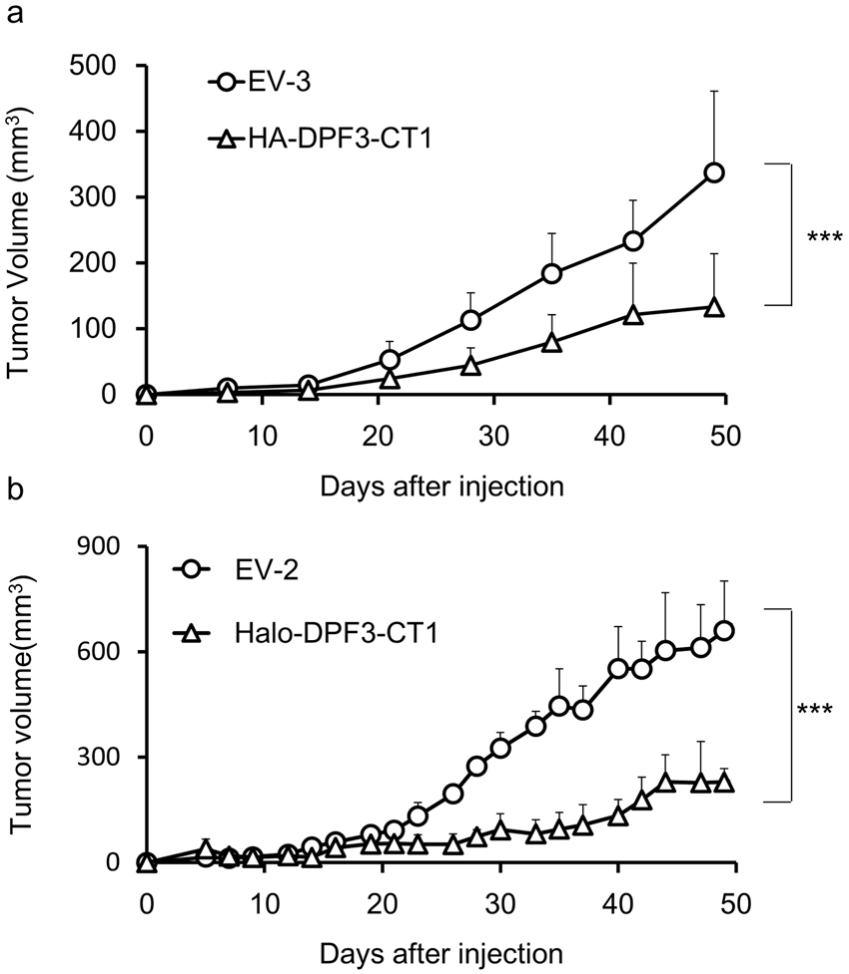
Tumorigenicity of A549 cells expressing DPF3-CT1. (**a**,**b**) Effects of HA-DPF3-CT1 (**a**) or Halo-DPF3-CT1 (**b**) for tumorigenicity using mouse xenograft models. A549 cells (1 × 10^6^ cells) transduced with lentivirus vectors expressing DPF3-CT1 or Empty Vector (Ctrl) at an MOI of 3.0 were injected into the left trunk of nude mice. Tumor volumes were measured and analyzed by two-way ANOVA with a Tukey post hoc test (***P < 0.001) and are represented as means ± SD ((**a**) *n* = 10, (**b**) *n* = 3).

HA-DPF3-CT1 expressing A549 cells were injected into immunodeficient mice to test the tumor-suppressing activity of CT1 *in vivo*. As shown in Fig. 6a, the onset of tumor formation was significantly delayed, and tumor growth was significantly reduced, by HA-DPF3-CT1 expression. Similar results were obtained when Halo-DPF3-CT1 was used (Fig. 6b). These results further indicate that the d4-family proteins are a promising target for future cancer therapies.

## Discussion

In our present study using epithelial tumor cells, we showed that the CT1 peptide comprising the 84 N-terminal amino acids of either the DPF2 or DPF3 protein functions as a dominant-negative mutant. CT1 inhibits SWI/SNF complex-dependent NF-κB transactivation (Figs 2 and 3) and anchorage-independent growth (Figs 1e and 4b), both of which require the endogenous expression of the d4-family proteins. Since our PLA results clearly showed that CT1 retains the full adaptor function of the parent d4-family proteins, linking the SWI/SNF complex and NF-κB dimers (Fig. 4D and E), the C-terminal region of d4-family protein that is lacked in CT1 would contain functional domain essential for SWI/SNF complex-dependent NF-κB transactivation.

When A549 cells expressing HA-DPF3a-CT1 or HA-DPF3a were analyzed by Chromatin immunoprecipitation (ChIP) assay, RelA is clearly recruited to the promoters of *IL6*, *IL8*, *TNF* and *ICAM1* genes after TNF-α treatment. But Brm, HA-CT1 and HA-DPF3a were detectable independent of TNF-α treatment (Supplementary Fig. S6) in these four promoters, consistent with our previous observations in 293FT cells^17^. Whereas SWI/SNF-dependent and independent promoters were not able to be discriminated by this ChIP analysis, these results suggest that CT1 behaves like its parental d4-family proteins concerning its recruitment to chromatin. Therefore, CT1 would probably perform its inhibitory function in the form of the SWI/SNF complex-CT1- NF-κB. This might lead to a hypothesis that the lack of C-terminal region is very critical for inhibitory function of CT1, which can be detectable even when CT1 is recruited to the promoters as a component of the SWI/SNF complex-CT1-NF-κB.

In the C-terminal region of DPF1, DPF2 and DPF3b, there is a tandem repeat of PHD fingers, which have been reported to bind acetylated^13^ or methylated^13^ or unmodified histone^41^. It is noteworthy that PHD fingers of DPF2 and MOZ have been recently reported to bind crotonylated histone (H3K14cr) at very low dissociation constants^22^. Although as histone readers in the SWI/SNF complex, the bromodomain in BRG1 or Brm has been intensively studied as a module that binds selected acetyl-lysine on histones^42,43^, the potent acetyl-lysine-competitive inhibitors failed to display any antiproliferative phenotype in many cancer cell lines^43^. Therefore, the crotonylated-lysine competitive inhibitors might be alternatively useful as anti-cancer drugs targeting epigenetical regulation by the SWI/SNF complex.

It is still largely unknown at this moment, how broadly the CT1 peptide can work on tumor cells. We expect that CT1 would not perform anti-tumor activity in cancers that lack both Brm and BRG1. Considering that activation of endogenous NF-κB activity has been observed in many epithelial tumors cells as well as melanomas and various leukemias and myelomas^1,2,4,5^, we believe CT1 would be effective to wide variety of tumors competent for the functional SWI/SNF complex. Since depletion of Brm in BRG1-deficient cancers, which are often detected in NSCLCs, has been shown to cause synthetic lethality^44^, CT1 might be highly effective in such SWI/SNF-dependent tumors. We expect that CT1 would not perform anti-tumor activity in such cancer cells like SW13 cells that lack both Brm and BRG1. In these cells, the loss of the functional SWI/SNF complex has been thought to be the causative of their tumorigenicity and therefore Brm and BRG1 are regarded as tumor suppressor genes in this context.

It is also worth noting that CT1 efficiently suppresses anchorage-independent growth without affecting the growth rate of monolayer cultures (Figs 1d,e and 4A,B). This might suggest that CT1 has no significant effect on non-cancerous cells. Given that CT1 suppresses only about 25% of NF-κB target genes, it is likely to be a powerful tool for experimentally dissecting and isolating the crucial NF-κB target genes required for oncogenic potential. In our present study, we identified *IL6* as an important CT1 target gene that contributes to anchorage-independent growth (Fig. 5c). This finding is consistent with previous reports that cell autonomous IL-6 production is crucial for the maintenance of transforming phenotypes and epithelial tumor cell growth in suspension^37^. Other clinical studies have also revealed increased serum IL-6 concentrations in patients with advanced stages of various cancers^39,45^.

Finally, CT1 expressing cells were shown to reduce tumorigenicity in a mouse xenograft model. Unlike the larger subtype of NF-κB target genes that are insensitive to CT1, CT1-sensitive genes would have less effect on the function of normal cells. Taken together, these results indicate that the d4-family proteins represent promising cancer therapy targets and that CT1 will be a viable and powerful starting point for the future development of new types of NF-κB inhibitors.

## Methods

### Cell culture and cell growth assays

The human cancer cell lines A549 (non-small-cell lung carcinoma; American Type Culture Collection), H1299 (non-small-cell lung carcinoma; American Type Culture Collection), HeLaS3 (cervical carcinoma; Cell Resource Center for Biomedical Research, Institute of Development, Aging and Cancer, Tohoku University, Japan), and MDA-MB-231 (human breast cancer; American Type Culture Collection) were used in the present study. The cell lines used in Supplementary Fig. S3 were previously described^12^. All cultures were maintained in Dulbecco’s modified Eagle’s medium containing 10% fetal calf serum. Cell growth in monolayer cultures was measured using CellTiter-Glo (Promega) in accordance with the manufacturer’s instructions. Anchorage-independent growth assays were performed as described previously^16^.

### Isolation of nuclear extracts and immunoprecipitation

Nuclear extracts were prepared from A549 or HeLeS3 cells using NE-PER Nuclear and Cytoplasmic Extraction Reagents (Pierce) in accordance with the manufacturer’s instructions and were finally diluted 8-fold in buffer [50 mM Tris-HCl (pH 7.5), 100 mM KCl, 1 mM DTT, 20% glycerol, and protease inhibitor cocktail (Nacalai Tesque)]. Immunoprecipitations were performed using Dynabeads Protein G (Invitrogen) with anti-HA antibody (#3724; Cell Signaling) according to the manufacturer’s instructions.

### DNA transfection and preparation of retro-/lentivirus

Lipofectamine 2000 (Invitrogen) was used for the transfection of plasmids into cell lines in accordance with the manufacturer’s instructions. The preparation and transduction of vesicular stomatitis virus-G (VSV-G) pseudotyped retrovirus and lentivirus vectors were performed as described previously^16,46^.

### Quantitative RT-PCR

Total RNA was extracted using a mirVana microRNA Isolation Kit (Ambion). To detect coding gene mRNAs, cDNA was synthesized with a PrimeScript™ RT Reagent Kit with cDNA Eraser (Perfect Real-Time; TaKaRa Bio) in accordance with the manufacturer’s instructions. qRT-PCR was performed using a SYBR® Select Master Mix (Applied Biosystems). *GAPDH* was used as an internal control. The primer pairs used are listed in Supplementary Table S3. PCR amplifications were performed in triplicate using a StepOne Plus Real-Time PCR system (Applied Biosystems).

### Western blotting

Total protein extracts were prepared by boiling the cells in SDS sample buffer for 10 min at 95 °C. The proteins were then separated by 10% SDS-PAGE and transferred onto Immobilon-P^SQ^ membranes (Millipore). Western blotting was performed by incubating the membranes overnight at 4 °C with primary antibodies against the following proteins: Brm (ab15597; Abcam), BRG1 (sc-10768; Santa Cruz), Halo tag (G928A; Promega), HA tag (#3724; Cell Signaling), and β-actin (sc-47778; Santa Cruz). After three washes with Tris-buffered saline containing Tween 20, the membranes were incubated with secondary antibodies [donkey anti-rabbit-horseradish peroxidase (AP182P) or donkey anti-mouse-horseradish peroxidase (AP192P); Millipore] for 1 h at room temperature. Signals were detected using ECL reagent (Promega) or ImmunoStar LD (Wako). Amounts of charged protein samples were roughly normalized to β-actin.

### *In situ* proximity ligation assay

Cell were seeded onto an 8-Well Lab-Tek II chamber slide (Nunc). The cells were then fixed with PBS containing 4% paraformaldehyde (Nacalai Tesque) for 10 min at room temperature (RT), washed twice with PBS and permeabilized with 0.2% Triton X-100 PBS for 10 min at RT. After washing twice with PBS, blocking was performed using a 1:1 mixture of 5% BSA, 0.02% NaN_3_ PBS and Blocking One (Nacalai Tesque) for 1 h at 37 °C. The samples were then incubated overnight at 4 °C with primary antibodies [anti-Brm (Rabbit), CST #11966; anti-RelA (Mouse), CST #6956; anti-p50 (Mouse), Santa Cruz #sc-8414; and anti-RelA (Rabbit), Santa Cruz #sc-372]. The PLA procedure was performed using the Duolink *In Situ* Starter Set ORANGR (Sigma) in accordance with the manufacturer’s instructions. Anti-mouse MINUS and anti-rabbit PLUS PLA probes were used. Fluorescence was detected using a florescence microscope (BZ-X710; Keyence).

### Microarray analysis

Total RNA was prepared using a *mir*Vana miRNA isolation kit (Ambion) and DNA was removed using a TURBO DNA-*free*™ *kit* (Ambion) according to the manufacturer’s instructions. The quality of all RNA samples was measured using a 2200 TapeStation (Agilent). RNA samples with RNA integrity numbers higher than 9.0 were used for gene expression analysis. Gene expression profiles were assessed using a SurePrint G3 Human Gene Expression v3 8 × 60 K Microarray kit (Agilent) according to the manufacturer’s protocol. Normalized expression data were calculated using R package. Microarray data analysis was supported by Cell Innovator (Fukuoka, Japan). All expression data were deposited in the Gene Expression Omnibus (GEO accession number GSE93770).

### Nude mouse xenograft model

A549 cells (1 × 10^6^ cells/50 μl in DMEM) transduced with lentivirus vectors expressing HA-DPF3-CT1 or empty vector (EV-3) at an MOI of 3.0 were sc injected into the left flank of 6-week-old female BALB/c nu/nu mice (CLEA Japan). Tumors were measured with digital calipers on the days indicated, and the tumor volumes were calculated as the length × width^2^/2 and represented as means ± SD (n = 10). All animal experimental protocols were performed in accordance with the policies of the Animal Ethics Committee of the University of Tokyo and performed in compliance with University’s Guidelines for the Care and Use of Laboratory Animals. All animal procedures were approved by the Animal Care and Use Committee of the University of Tokyo.

### Statistical analysis

The results of qRT-PCR and colony formation in soft agar are represented as means ± SD. Signal numbers from *in situ* PLA are represented as means ± SEM. The statistical significance of the data obtained from qRT-PCR assays and anchorage-independent growth assays was determined using a two-tailed unpaired Student’s *t*-test. Tumor volumes were analyzed by two-way ANOVA with a Tukey post hoc test. P < 0.05 was considered statistically significant.

## Electronic supplementary material

Supplementary information
