# Supplementary material for: Tumor suppression via inhibition of SWI/SNF complex-dependent NF-κB activation
Source: Sci Rep. 2017 Sep 18;7:11772. doi: 10.1038/s41598-017-11806-9 (PMC5603518; doi:10.1038/s41598-017-11806-9)
Supplement: Supplementary file 1 — Supplementary information [file 41598_2017_11806_MOESM1_ESM.pdf]

Supplementary information

Supplementary Methods

Supplementary References

Supplementary Table S1-3

Supplementary Figure S1-6

Tumor suppression via inhibition of SWI/SNF complex-dependent NF-κB activation

Kazuyoshi Kobayashi[1,2,3], Hiroaki Hiramatsu[1,2,3], Shinya Nakamura[1], Kyousuke Kobayashi[1], Takeshi Haraguchi[1,2] & Hideo Iba[1,2,4]

[1]Division of Host-Parasite Interaction, Department of Microbiology and Immunology, The Institute of Medical Science, The University of Tokyo, Tokyo 108-8639, Japan

[2]Division of RNA Therapy, Medical Mycology Research Center, Chiba University, Chiba 260-8673, Japan

[3]These authors contributed equally to this work.

[4]To whom correspondence should be addressed.

Tel.: 81-43-226-2801; Fax: 81-43-226-2486; E-mail: iba@ims.u-tokyo.ac.jp

Supplementary Methods

Plasmid preparation for retro/lentivirus vectors

Pairs of oligonucleotides encoding gene-specific short hairpin RNA (shRNA) were synthesized (listed in Supplementary Table S3) and inserted into the BbsI/EcoRI sites of pmU6 [1]. The pmU6 derivatives shCre#4 (used as a negative control) and shBrm#4 were previously described[2,3]. These pmU6-based plasmids were doubly digested with BamHI and EcoRI, and inserted into the same site of pSSSP (EV-1)[3] to generate retrovirus vector plasmids. To generate the *IL6* shRNA vector, the pmU6-based plasmid was digested with BamHI and EcoRI, and inserted into the same site of pLSP [4]. For construction of the Halo-tagged CT1 expression vectors, the SgfI/PmeI fragment of pFN21K Halo tag CMV Flexi Vector (Promega) encoding barnase was replaced with DNA fragments encoding multi cloning sites (MCS) or DPF2-CT1, which contains the terminal sequences of SgfI/PmeI (synthesized by Genscript, listed in Supplementary Table S3) to generate pFN21K-Halo-MCS and pFN21K-Halo-DPF2-CT1, respectively. Halo-MCS and Halo-DPF2-CT-containing fragments were isolated by doubly

digesting these plasmids with NheI and BamHI, and replaced with the SpeI/BamHI fragment of pXL001 (26112; Addgene), which encodes tTR-KRABi to generate pXL001-Halo-MCS (EV-2) and pXL001-Halo-DPF2-CT1.

For the construction of pXL001-Halo-DPF3-CT1, the DPF3-CT1 fragment (about 250 bp) prepared by PCR with a primer pair containing the SgfI or PmeI site (Supplementary Table S3), was ligated with the SgfI/PmeI digest of pXL001-Halo-MCS. For HA-tagged vector construction, synthetic oligonucleotides encoding the HA sequence (Supplementary Table S3) were inserted into the BamHI/EcoRI site of pcDNA3.1(+) (Invitrogen) to generate pcDNA3.1(+)-HA-MCS. DPF3a, DPF3b and DPF3-CT1 fragments were obtained by PCR using the primer sets listed in Supplementary Table S3, digested with EcoRI and EcoRV, and then inserted into the same site of pcDNA3.1(+)-HA-MCS to generate pcDNA3.1(+)-HA-DPF3a, pcDNA3.1(+)-HA-DPF3b and pcDNA3.1(+)-HA-DPF3-CT1, respectively. DNA fragments for the other truncation mutants of DPF3 (Δ1-39, Δ1-32, Δ1-8, Δ40-76 and Δ53-76) were synthesized (Supplementary Table S3), digested with EcoRI and EcoRV, and inserted into the same site of pcDNA3.1(+)-HA-

MCS. The DNA fragments encoding HA-tagged proteins were then obtained by digestion with NheI and BglII and inserted into the SpeI/BamHI site of pXL001 to generate pXL001-HA (EV-3), pXL001-HA-DPF3a, pXL001-HA-DPF3b, pXL001-HA-DPF3-CT1, as well as, pXL001-HA-DPF3-Δ1-39, -Δ1-32, -Δ1-8, -Δ40-76 and -Δ53-76.

For cDNA expression, the *EF1α* promoter region was amplified by PCR from pXL001 using the primer sets listed in Supplementary Table S3 and digested with NheI. pLSP was digested with ClaI, blunt ended using T4 DNA polymerase and then digested with XbaI. The resulting 1.5 kb and 5.1 kb fragments were ligated to generate pLE. Pairs of oligonucleotides containing multi cloning sites (MCS) were synthesized as listed in Supplementary Table S3 and inserted into the EcoRV/ClaI site of pLE to generate pLE-MCS. The IRES-Puro^r fragment was obtained by PCR from pMXs-IP [5], using primer sets listed in Supplementary Table S3, and digested with XbaI and ClaI. The resulting 1.2 kb fragment was inserted into the XbaI/ClaI site of pLE-MCS to generate pLE-IP (EV-4). Pairs of oligonucleotides encoding a 3×FLAG tag were synthesized as listed in Supplementary Table S3, annealed, extended, digested with BglII and MfeI, and inserted into the same site in the

MCS of pLE-IP. Brm and BRG1 fragments were amplified by PCR using the primer sets listed in Supplementary Table S3 and cloned into the BamHI/EcoRI site of pCR2.1 (Invitrogen). Brm fragment was digested with EcoRI and XhoI, and inserted into the same site in MCS of pLE-IP to generate pLE-Brm-IP. BRG1 fragment was digested with MfeI and XbaI, and inserted into the same site in MCS of pLE-IP to generate pLE-BRG1-IP. All plasmids were confirmed by DNA sequencing.

Chromatin Immunoprecipitation (ChIP)

ChIP assays were performed using SimpleChIP Enzymatic Chromatin IP Kit (Cell Signaling) according to the manufacture's instructions. Briefly, A549 cells were cross-linked with 1% formaldehyde at room temperature (RT) for 10 min. Chromatin fragments were incubated overnight on a rotating platform at 4 °C with the following antibodies (2µg of each): normal rabbit IgG (#2729; Cell Signaling), anti-Brm (ab15597; Abcam), anti-RelA (SC-372; SantaCruz) and anti-HA tag (#3724; Cell Signaling). The specific primer pairs used in this assay are listed in supplemental Table S3.

Supplementary References

1 Yu, J. Y., DeRuiter, S. L. & Turner, D. L. RNA interference by expression of short-interfering RNAs and hairpin RNAs in mammalian cells. *Proc Natl Acad Sci U S A* **99**, 6047-6052, doi:10.1073/pnas.092143499 (2002).

2 Haraguchi, T. *et al.* SiRNAs do not induce RNA-dependent transcriptional silencing of retrovirus in human cells. *FEBS Lett* **581**, 4949-4954, doi:10.1016/j.febslet.2007.09.028 (2007).

3 Yamamichi, N. *et al.* The Brm gene suppressed at the post-transcriptional level in various human cell lines is inducible by transient HDAC inhibitor treatment, which exhibits antioncogenic potential. *Oncogene* **24**, 5471-5481, doi:10.1038/sj.onc.1208716 (2005).

4 Haraguchi, T., Ozaki, Y. & Iba, H. Vectors expressing efficient RNA decoys achieve the long-term suppression of specific microRNA activity in mammalian cells. *Nucleic Acids Res* **37**, e43, doi:10.1093/nar/gkp040 (2009).

5 Kitamura, T. *et al.* Retrovirus-mediated gene transfer and expression cloning: powerful tools in functional genomics. *Exp Hematol* **31**, 1007-1014 (2003).

Supplementary Table S1

CT1-sensitive and CT1-insensitive genes in A549 and HeLaS3 cells categorized by microarray analysis.

No.	Gene	Induction					CT1-sensitivity				CpG size	CpG island
		A549		HeLaS3			A549		HeLaS3			
		ratio	Z-score	ratio	Z-score		ratio	Z-score	ratio	Z-score		
1	CCL20	646.53	15.31	80.63	11.63	CT1-sensitive	0.40	-3.47	0.34	-4.54	-	-
2	CXCL1	151.01	20.02	6.06	4.80		0.65	-2.74	0.26	-2.76	626	+
3	CXCL8 (IL8)	122.23	17.32	26.98	14.11		0.42	-3.24	0.29	-5.30	-	-
4	DUSP5	2.09	3.16	5.92	10.12		0.52	-2.49	0.59	-2.28	1521	+
5	IL6	74.61	10.20	12.27	13.11		0.09	-7.24	0.37	-4.40	-	-
6	INHBA	16.98	2.83	9.39	9.59		0.38	-2.10	0.44	-2.86	284	-
7	BCL2A1	3.72	4.75	0.88	-0.75		0.47	-2.85	0.71	-1.48	-	-
8	CSF2	97.69	10.84	NULL	NULL		0.30	-4.57	NULL	NULL	-	-
9	CXCL5	2.86	5.29	1.19	0.77		0.37	-4.66	0.95	-0.17	216	-
10	IL1B	18.80	6.94	NULL	NULL		0.13	-4.45	NULL	NULL	-	-
11	TRAF1	2.43	3.21	NULL	NULL		0.45	-2.34	NULL	NULL	904	+
12	AKR1C1	1.01	0.06	2.28	2.21		0.49	-4.72	0.06	-2.34	-	-
13	DPYD	1.12	0.27	25.20	3.52		0.65	-0.95	0.04	-2.59	535	+
14	BCL3	2.88	5.33	2.23	5.10	CT1-insensitive	1.36	2.18	0.90	-0.51	563	+
15	CCL2	7.14	9.89	5.38	9.58		0.74	-1.93	1.47	1.97	-	-
16	CD83	10.75	10.12	2.41	5.00		0.80	-0.72	0.92	-0.43	590	+
17	EBI3	3.58	4.61	2.83	2.79		2.01	2.66	2.79	2.11	-	-
18	ICAM1	28.45	12.07	3.89	7.10		1.10	0.44	1.09	0.38	1678	+
19	IER2	1.97	3.47	3.12	7.62		1.25	1.62	1.15	1.30	4362	+
20	IL1A	23.11	3.13	11.14	2.66		0.42	-1.87	0.78	-0.51	-	-
21	IL23A	4.53	5.46	3.22	3.13		1.15	0.32	0.47	-1.55	-	-
22	IRF1	19.93	12.74	4.77	8.17		1.34	1.26	0.70	-1.50	1093	+
23	JUNB	3.26	4.27	4.65	8.04		1.00	0.00	1.44	1.52	4550	+
24	KDM6B	8.04	4.93	4.89	4.23		11.88	5.39	5.74	3.79	3458	+
25	NFKB1	1.83	2.95	2.42	5.03		0.97	-0.11	0.81	-0.95	1138	+
26	NFKB2	2.09	3.16	1.96	3.53		0.99	-0.01	0.90	-0.43	1828	+
27	NFKBIA	12.28	12.86	5.03	10.83		0.94	-0.25	1.01	0.05	943	+
28	NFKBIE	2.27	4.01	2.99	6.22		1.01	0.12	0.80	-0.97	567	+
29	NR4A2	3.31	2.83	4.52	6.47		1.54	0.92	2.29	3.50	1278	+
30	NUAK2	2.84	5.25	2.77	6.47		1.03	0.31	0.92	-0.42	803	+
31	PIM1	4.17	6.96	2.56	5.34		1.35	1.31	1.06	0.29	2364	+
32	PLK3	2.14	3.71	1.71	3.04		0.78	-1.05	0.69	-1.63	1249	+
33	REL	2.65	3.53	1.70	2.77		1.58	1.75	0.96	-0.20	891	+
34	RELB	1.81	2.98	2.62	6.13		0.99	0.04	1.12	0.60	787	+
35	SAT1	2.13	3.80	2.34	4.83		0.96	-0.13	1.29	1.13	570	+
36	SDC4	3.42	6.01	4.84	10.02		1.16	0.66	1.16	0.91	709	+
37	SOD2	2.41	4.30	2.27	5.21		0.90	-0.41	0.68	-1.95	1306	+
38	STAT5A	4.49	5.43	1.94	3.47		0.85	-0.58	0.96	-0.15	826	+
39	TICAM1	1.81	2.90	1.57	3.04		0.94	-0.24	1.11	0.67	459	+
40	TNF	622.64	15.22	31.54	14.78		0.84	-0.69	1.12	0.49	-	-
41	BDKRB1	3.01	4.71	NULL	NULL		0.77	-0.96	NULL	NULL	-	-
42	CCL17	6.29	4.35	NULL	NULL		0.63	-1.01	NULL	NULL	-	-
43	CCL5	7.03	4.61	1.29	1.08		1.29	0.78	1.80	2.05	-	-
44	CD40	2.79	2.42	NULL	NULL		1.26	0.69	NULL	NULL	238	-
45	CFB	3.11	2.68	NULL	NULL		0.77	-0.78	NULL	NULL	-	-
46	CSF1	3.16	4.16	1.86	1.69		4.48	3.26	0.91	-0.17	746	+
47	FSTL3	1.63	2.47	1.40	2.13		0.75	-1.32	1.13	0.75	942	+
48	LTB	10.34	8.43	NULL	NULL		1.44	1.40	NULL	NULL	-	-
49	NFKBIZ	5.67	4.10	2.03	1.92		0.86	-0.43	1.12	0.23	1291	+
50	ORM1	2.06	2.61	0.72	-1.41		0.68	-1.15	0.88	-0.44	-	-

51	PDGFB	2.09	2.68	0.91	-0.41	0.72	-0.99	0.47	-2.58	2697	+
52	SELE	19.76	2.97	1.69	1.43	0.48	-1.59	0.29	-1.00	-	-
53	SERPINA3	9.72	9.69	0.98	-0.10	1.08	0.34	1.39	1.16	-	-
54	SOX9	2.34	2.01	NULL	NULL	1.17	0.49	NULL	NULL	4168	+
55	TNFRSF9	3.82	3.17	NULL	NULL	0.60	-1.51	NULL	NULL	291	-
56	TNIP1	1.61	2.40	1.44	2.07	0.85	-0.70	1.13	0.54	778	+
57	AQP4	NULL	NULL	6.27	2.05	NULL	NULL	0.16	-1.49	210	-
58	BMP2	3.90	1.38	4.25	8.23	0.56	-0.66	0.75	-1.44	1494	+
59	CD274	NULL	NULL	3.00	4.71	NULL	NULL	1.20	0.76	220	-
60	CXCR1	NULL	NULL	6.79	2.13	NULL	NULL	1.11	0.21	-	-
61	DUSP1	1.42	1.73	1.90	3.35	0.87	-0.58	1.44	1.59	2124	+
62	EDN1	1.04	0.20	2.20	4.49	1.13	0.54	0.92	-0.34	-	-
63	ELF3	1.39	1.60	2.00	3.94	1.31	1.36	1.14	0.56	-	-
64	GADD45B	1.15	0.70	1.85	3.51	1.06	0.29	2.15	3.36	2218	+
65	OLR1	3.15	1.17	4.91	4.24	0.21	-1.70	0.49	-1.48	-	-
66	PTGES	1.32	1.03	2.81	2.77	1.41	1.04	1.56	1.54	-	-
67	SAA1	1.79	1.37	4.24	6.19	0.66	-1.23	1.21	0.67	-	-
68	SAA2	1.65	1.18	4.52	4.02	0.75	-0.63	1.36	1.07	-	-
69	TAPBP	1.53	1.55	1.66	2.19	1.51	1.24	1.18	0.59	715	+
70	THBS1	0.70	-0.86	2.66	4.19	2.26	1.74	0.94	-0.29	1040	+
71	TNFSF10	NULL	NULL	10.32	2.58	NULL	NULL	0.10	-1.87	-	-
72	IL32	3.12	4.86	2.56	4.03	0.55	-2.26	1.64	2.07		-
73	PRDM1	2.94	3.90	2.14	3.98	0.33	-3.29	0.74	-1.27	249	-
74	CXCL2	32.89	14.87	6.59	9.86	0.84	-0.75	0.33	-4.72	623	+
75	CXCL3	65.47	15.08	13.39	11.11	0.64	-1.89	0.17	-6.07	636	+
76	EGR1	58.08	14.64	6.10	9.45	2.26	3.49	0.50	-2.92	2638	+
77	F3	2.10	2.68	1.62	2.52	0.79	-0.68	0.54	-2.57	802	+
78	IER3	3.76	6.67	4.36	9.35	1.00	0.14	0.55	-3.02	2133	+
79	KLF10	2.13	3.23	2.51	4.81	1.17	0.71	0.59	-2.30	2704	+
80	PI3	6.49	4.42	3.67	5.57	0.59	-1.56	0.32	-3.97		-
81	PLAU	4.99	8.24	3.39	6.94	0.78	-1.55	0.60	-2.62	683	+
82	PTGS2	8.43	7.69	8.51	5.69	0.63	-1.72	0.02	-3.19	270	-
83	PTX3	53.83	9.43	7.96	10.84	0.91	-0.34	0.26	-5.74	498	+
84	TNFAIP2	3.11	5.82	4.77	10.47	0.88	-1.08	0.62	-3.00	689	+
85	TNFAIP3	38.11	15.50	11.42	13.85	45.45	16.05	0.42	-3.80	889	+

All the NF-κB target genes that are induced by TNF-α in either A549 or HeLaS3 cells were extracted from the heat map shown in Fig 2a. We selected TNF-α–inducible genes using the following criteria: expression ratio of TNF-α–treated EV-1–transduced cells to untreated EV-1–transduced cells > 1.5 and a Z-score > 2 (marked in dark grey). CT1-sensitive genes were further selected by the following criteria: ratio of CT1-expressing cells to EV-2–transduced cells < 0.66 and a Z-score < –2 (marked in light grey). The other genes were classified as CT1-insensitive. The CpG islands size of each gene promoter was evaluated by UCSC genome browser and the promoters whose CpG islands size is larger than 300 bp was considered as CpG islands (+).

1–13, Genes that are both TNF-α inducible and CT1 sensitive in both A549 and HeLaS3 cells are marked in green. Genes that are both TNF-α inducible and CT1 sensitive in A549 cells alone were marked in yellow, whereas those in HeLaS3 cells alone were marked in pink. **14–71**, CT1-insensitive genes induced by TNF-α in both A549 and HeLaS3 cells (light blue), in A549 cells alone (orange), or in HeLaS3 cells (red). **72-85**, Genes whose CT1 sensitivity are differently categorized between A549 and HeLaS3 cells (white). These genes are not considered in Table 1.

Supplementary Table S2.
Biological activity of deletion mutants derived from HA-DPF3-CT1 in A549 cells.

Vector	Protein expression[a]	Induction after TNF-α stimulation[b] (%)		Suppression of AIG[c] (%)
		IL6	*IL8*	
CT1	+	21 ± 1.9	33 ± 9.8	5.7 ± 4.1
Δ1-39	-	92 ± 2.5	110± 27	110 ± 8.2
Δ1-32	-	100 ± 6.2	95 ± 12	121 ± 2.1
Δ1-8	+	38 ± 1.9	41 ± 2.7	25 ± 8.7
Δ40-76	+	85 ± 4.3	63 ± 1.9	78 ± 11
Δ53-76	+	95 ± 3.0	80 ± 13	96 ± 9.6

a: Protein expression was evaluated using immunocytochemical staining with anti-HA antibody.
b: After 1h of treatment with TNF-α, the mRNA levels of *IL6* and *IL8* genes in A549 cells transduced with CT1 derivatives were examined by qRT-PCR and are shown as the percentage of the levels in A549 cells transduced with EV-3.
c: Relative colony formation was evaluated as shown in Figure 1e.

Supplementary Table S3.

Oligonucleotides used in this study.

● Oligonucleotides used for shRNA expression vectors

shIL-6#1-sense	5'-TTTGTATTTATATAATGTATAAATGCTTCCTGTCACATTTATACATTATATAAATACTTTTTG-3'
shIL-6#1-antisense	5'-AATTCAAAAAGTATTTATATAATGTATAAATGTGACAGGAAGCATTTATACATTATATAAATA-3'
shDPF1-3'UTR#2-sense	5'-TTTGTTAATATATACAAAGAGTCCGCTTCCTGTCACGGACTCTTTGTATATATTAACTTTTTG-3'
shDPF1-3'UTR#2-antisense	5'-AATTCAAAAAGTTAATATATACAAAGAGTCCGTGACAGGAAGCGGACTCTTTGTATATATTAA-3'
shDPF1-3'UTR#4-sense	5'-TTTGAATTAACTTGTTCTGTGTATGCTTCCTGTCACATACACAGAACAAGTTAATTCTTTTTG-3'
shDPF1-3'UTR#4-antisense	5'-AATTCAAAAAGAATTAACTTGTTCTGTGTATGTGACAGGAAGCATACACAGAACAAGTTAATT-3'
shDPF2-3'UTR#3-sense	5'-TTTGTAGCTTCACCTTGTTATTCCGCTTCCTGTCACGGAATAACAAGGTGAAGCTACTTTTTG-3'
shDPF2-3'UTR#3-antisense	5'-AATTCAAAAAGTAGCTTCACCTTGTTATTCCGTGACAGGAAGCGGAATAACAAGGTGAAGCTA-3'
shDPF2-3'UTR#4-sense	5'-TTTGCTCTTAACTGAATTGGGAGCGCTTCCTGTCACGCTCCCAATTCAGTTAAGAGCTTTTTG-3'
shDPF2-3'UTR#4-antisense	5'-AATTCAAAAAGCTCTTAACTGAATTGGGAGCGTGACAGGAAGCGCTCCCAATTCAGTTAAGAG-3'
shDPF2-3'UTR#6-sense	5'-TTTGGTGATCACAGGGTTCAAACAGCTTCCTGTCACTGTTTGAACCCTGTGATCACCTTTTTG-3'
shDPF2-3'UTR#6-antisense	5'-AATTCAAAAAGGTGATCACAGGGTTCAAACAGTGACAGGAAGCTGTTTGAACCCTGTGATCAC-3'
shDPF3a-CDS#2-sense	5'-TTTGCTTTGATGAGGACGATTTGGGCTTCCTGTCACCCAAATCGTCCTCATCAAAGCTTTTTG-3'
shDPF3a-CDS#2-antisense	5'-AATTCAAAAAGCTTTGATGAGGACGATTTGGGTGACAGGAAGCCCAAATCGTCCTCATCAAAG-3'
shDPF3a-3'UTR#4-sense	5'-TTTGAAATCGAAGCAATATCCTGTGCTTCCTGTCACACAGGATATTGCTTCGATTTCTTTTTG-3'
shDPF3a-3'UTR#4-antisense	5'-AATTCAAAAAGAAATCGAAGCAATATCCTGTGTGACAGGAAGCACAGGATATTGCTTCGATTT-3'

shDPF3b-CDS#6-sense	5'-TTTGGGAACTGCTCAAAGAGAAAGGCTTCCTGTCACCTTTCTCTTTGAGCAGTTCCCTTTTTTG-3'
shDPF3b-CDS#6-antisense	5'-AATTCAAAAAAGGGAACTGCTCAAAGAGAAAGGTGACAGGAAGCCTTTCTCTTTGAGCAGTTCC-3'
shDPF3b-CDS#7-sense	5'-TTTGATGACCAGCTACTCTTCTGCGCTTCCTGTCACGCAGAAGAGTAGCTGGTCATCTTTTTTG-3'
shDPF3b-CDS#7-antisense	5'-AATTCAAAAAAGATGACCAGCTACTCTTCTGCGTGACAGGAAGCGCAGAAGAGTAGCTGGTCAT-3'
shBrm#4-sense	5'-TTTGAATGTGGTGTTGGTGCTTTCGCTTCCTGTCACGAAAGCACCAACACCACATTCTTTTTTG-3'
shBrm#4-antisense	5'-AATTCAAAAAAGAATGTGGTGTTGGTGCTTTCGTGACAGGAAGCGAAAGCACCAACACCACATT-3'
shBrm#8-sense	5'-TTTGTGATAAACTACAAAGATAGGGCTTCCTGTCACCCTATCTTTGTAGTTTATCACTTTTTTG-3'
shBrm#8-antisense	5'-AATTCAAAAAAGTGATAAACTACAAAGATAGGGTGACAGGAAGCCCTATCTTTGTAGTTTATCA-3'
shBRG1-CDS#2-sense	5'-TTTGTTGGAAGTACATGATTGTGGGCTTCCTGTCACCCACAATCATGTACTTCCAACTTTTTTG-3'
shBRG1-CDS#2-antisense	5'-AATTCAAAAAAGTTGGAAGTACATGATTGTGGGTGACAGGAAGCCCACAATCATGTACTTCCAA-3'
shBRG1-CDS#4-sense	5'-TTTGCGTATCGCGGCTTTAAATACGCTTCCTGTCACGTATTTAAAGCCGCGATACGCTTTTTTG-3'
shBRG1-CDS#4-antisense	5'-AATTCAAAAAAGCGTATCGCGGCTTTAAATACGTGACAGGAAGCGTATTTAAAGCCGCGATACG-3'

● Oligonucleotides used for the preparation of Halo-tag expression vectors

MCS (Halo)-sense	5'-CGCCATGGAATTCAACGCGATCGCCTAGATATCGGTTTAAACCATCTCGAGCATGCGGCCGCATGTTT-3'
MCS (Halo)-antisense	5'-AAACATGCGGCCGCATGCTCGAGATGGTTTAAACCGATATCTAGGCGATCGCGTTGAATTCCATGGCGAT-3'
DPF2-CT1	5'-GCGATCGCCATGGCGGCTGTGGTGGAGAATGTAGTGAAGCTCCTTGGGGAGCAGTACTACAAAGATGCCATGGAGCAGTGCCACAATTACAATGCTCGCCTCTGTGCTGAGCGCAGCGTGCGCCTGCCTTTCTTGGACTCACAGACCGGAGTAGCCCAGAGCAATTGTTACATCTGGATGGAAAAGCGACACCGGGGTCCAGGATTGGCCTCCGGACAGCTGTACTCCTACCCTGCCCGG

	CGCTGGCGGAAAAAGCGGCGAGTTTAAAC-3’
DPF3-CT1-Fwd	5’-GCGATCGCCATGGCGACTGTCATTCACAACC-3’
DPF3-CT1-Rev	5’-GTTTAAACTCGTCTCTTCTTGCGCCAG-3’

● Oligonucleotides used for HA-tag expression vectors

HA-Tag-sense	5’-GATCCACTACCATGTACCCATACGATGTTCCAGATTACGCTG-3’
HA-Tag-antisense	5’-AATTCAGCGTAATCTGGAACATCGTATGGGTACATGGTAGTG-3’
DPF3a/3b/3-CT1 (HA)-Fwd	5’-CGGAATTCGCGACTGTCATTCACAACCC-3’
DPF3a-Rev	5’-CGATATCAGATCTTTAAACGCAACTGCCCTTTTTATCTCTGTGGGC-3’
DPF3b-Rev	5’-CGATATCAGATCTTTAAACGGCCTGGCAGCCAAAGGCTGA-3’
DPF3-CT1-Rev	5’-CGATATCAGATCTTTAAACTCGTCTCTTCTTGCGCCAGCAGCGG-3’
DPF3-CT1 (Δ1-39)	5’-GAATTCCCCTTCCTGGACTCACAGACTGGGGTGGCCCAGAACAACTGCTACATCTGGATGGAGAAGAGGCACCGAGGCCCAGGCCTTGCCCCGGGCCAGCTGTATACATACCCTGCCCGCTGCTGGCGCAAGAAGAGACGAGTTTAAAGATCTGATATC-3’
DPF3-CT1 (Δ1-32)	5’-GAATTCGCAGAGCGCAGCGTGCGTCTTCCCTTCCTGGACTCACAGACTGGGGTGGCCCAGAACAACTGCTACATCTGGATGGAGAAGAGGCACCGAGGCCCAGGCCTTGCCCCGGGCCAGCTGTATACATACCCTGCCCGCTGCTGGCGCAAGAAGAGACGAGTTTAAAGATCTGATATC-3’
DPF3-CT1 (Δ1-8)	5’-GAATTCCTGAAAGCGCTCGGGGACCAGTTCTACAAGGAAGCCATTGAGCACTGCCGGAGTTACAACTCACGGCTGTGTGCAGAGCGCAGCGTGCGTCTTCCCTTCCTGGACTCACAGACTGGGGTGGCCCAGAACAACTGCTACATCTGGATGGAGAAGAGGCACCGAGGCCCAGGCCTTGCCCCGGGCCAGCTGTATACATACCCTGCCCGCTGCTGGCGCAAGAAGAGACGAGTTTAAAGATCTGATATC-3’
DPF3-CT1 (Δ40-76)	5’-GAATTCGCGACTGTCATTCACAACCCCCTGAAAGCGCTCGGGGACCAGTTCTACAAGGAAGCCATTGAGCACTGCCGGAGTTACAACTCACGGCTGTGTGCAGAGCG

	CAGCGTGCGTCTTCGCTGCTGGCGCAAGAAGAGACG AGTTTAAAGATCTGATATC-3’
DPF3-CT1 (Δ53-76)	5’-GAATTCGCGACTGTCATTCACAACCCCCTGAAAGC GCTCGGGGACCAGTTCTACAAGGAAGCCATTGAGCA CTGCCGGAGTTACAACTCACGGCTGTGTGCAGAGCG CAGCGTGCGTCTTCCCTTCCTGGACTCACAGACTGG GGTGGCCCAGAACAACCGCTGCTGGCGCAAGAAGA GACGAGTTTAAA GATCTGATATC-3’

● Oligonucleotides used for Brm and BRG1 expression vectors

EF1α-Fwd	5’-GTTTAAACGCCACAAATGGCAGTATTCATCCA-3’
EF1α-Rev	5’-AAAGCTAGCATCGATGATATCCTCACGACACCTGAAATG GAAGA-3’
MCS (pLE)-sense	5’-ATCAGATCTCAATTGCTCGAGGCGGCCGCCAGCTGTCTA GACAT-3’
MCS (pLE)-antisense	5’-CGATGTCTAGACAGCTGGCGGCCGCCTCGAGCAATTGAG ATCTGAT-3’
IRES-Puro^r-Fwd	5’-AAATCTAGACGGCCGCTACGTAAATTCCG-3’
IRES-Puro^r-Rev	5’-AAAATCGATGCTCGATCAGGCACCGGGCTTGCGGGT-3’
3×FLAG-sense	5’-AAAAAGATCTACTACCATGGACTACAAAGACCATGACGG TGATTATAAAGATCATGACAT-3’
3×FLAG-antisense	5’-TTTTCAATTGCTTGTCATCGTCATCCTTGTAGTCGATGTCA TGATCTTTATAATCACCGT-3’
Brm-Fwd	5’-GAATTCATGTCCACGCCCACAGACCC-3’
Brm-Rev	5’-CTCGAGTCACTCATCATCCGTCCCAC-3’
BRG1-Fwd	5’-CAATTGATGTCCACTCCAGACCCA-3’
BRG1-Rev	5’-TCTAGAGTCAGTCTTCTTCGCTGCCA-3’

● Primer pairs used for qRT-PCR

IL6-Fwd	5’-AGTAACATGTGTGAAAGCAGCAA-3’
IL6-Rev	5’-AAACTCCAAAAGACCAGTGATGA-3’
IL8-Fwd	5’-GGTGCAGTTTTGCCAAGGAG-3’
IL8-Rev	5’-TTCCTTGGGGTCCAGACAGA-3’
TNF-Fwd	5’-CCCCCCGAGTGACAAGCCTGTAG-3’
TNF-Rev	5’-TGAGGTACAGGCCCTCTGAT-3’

ICAM1-Fwd	5’-AACCTCAGCCTCGCTATGG-3’
ICAM1-Rev	5’-ACTTTTGAGGGGGACACAGAT-3’
DPF1-Fwd	5’-CCGGAAGGGAGCTGGA-3’
DPF1-Rev	5’-CAGGTAGGCGAGCACCAC-3’
DPF2-Fwd	5’-GAGATGTAGTGAAGC-3’
DPF2-Rev	5’-GCTACTCCGGTCTGTG-3’
DPF3a-Fwd	5’-TCAGACAACACAGGAGCCAG-3’
DPF3a-Rev	5’-AACTGAGGCCATTCCCAAGG-3’
DPF3b-Fwd	5’-AGCTACTCTTCTGCGATGACTG-3’
DPF3b-Rev	5’-TTCTCTTTGAGCAGTTCCCAGC-3’
Brm-Fwd	5’-CAGAAGCAGAGCCGCATCA-3’
Brm-Rev	5’-GGCCTGAAGTCTGTATTCC-3’
BRG1-Fwd	5’-AGATGTCTTCCGGGCCA-3’
BRG1-Rev	5’-AGCTGGTTCTGGTTAAATGGG-3’
GAPDH-Fwd	5’-CTCTGCTCCTCCTGTTCGAC-3’
GAPDH-Rev	5’-TTAAAAGCAGCCCTGGTGAC-3’
IL6 promoter-Fwd	5’-GTCTTGCCATGCTAAAGGACG-3’
IL6 promoter-Rev	5’-GCCTCAGACATCTCCAGTCC-3’
IL8 promoter-Fwd	5’- TCGTCATACTCCGTATTTGAT-3’
IL8 promoter-Rev	5’- AGAGAACTTATGCACCCTCA-3’
TNF promoter-Fwd	5’-TGCTTGTGTGTCCCCAACTT-3’
TNF promoter-Rev	5’- CTGGTCCTCTGCTGTCCTTG-3’
ICAM1 promoter-Fwd	5’-AGCGCGGTGTAGACCGTGATTCAA-3’
ICAM1 promoter-Rev	5’-GCTGGCCGCTTCAGCTCCGGAAT-3’

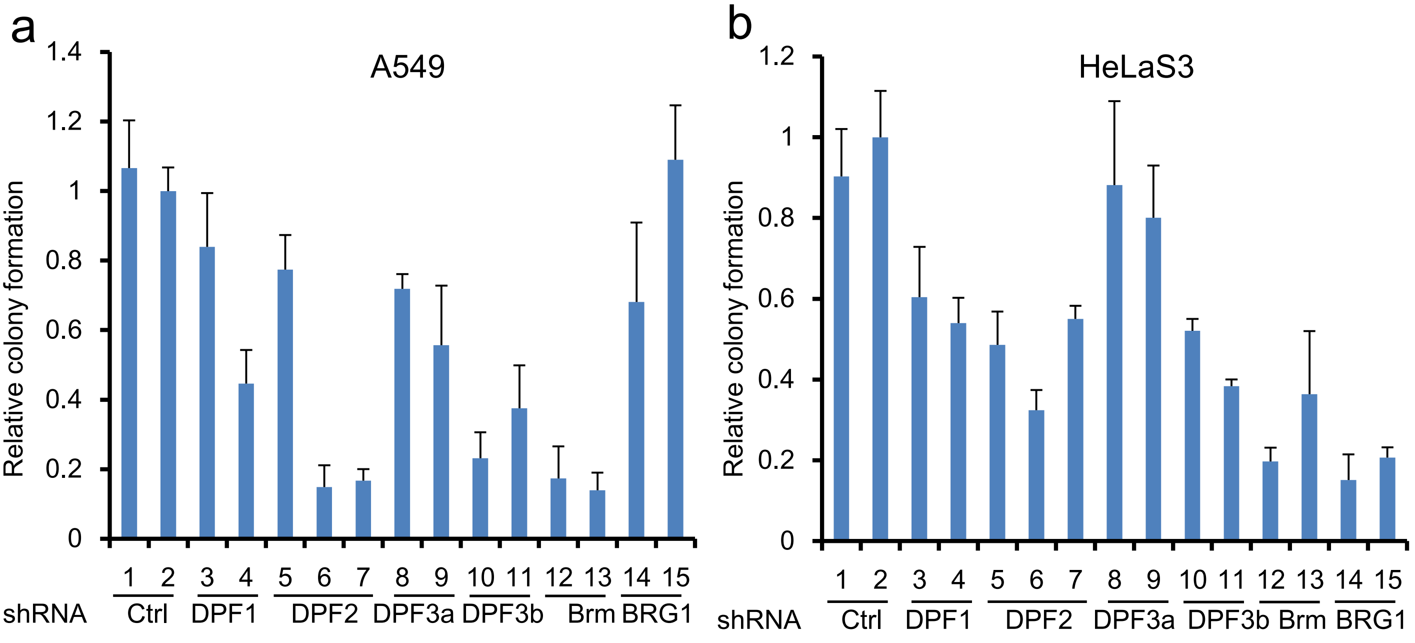

Supplementary Figure S1: Knockdown effects of each d4-family protein, Brm, and BRG1 on the anchorage-independent growth.

(a, b) HeLaS3 (a) or A549 (b) cells transduced with shRNA expressing lentivirus vectors were grown in soft agar. Colonies of more than 100 nm in diameter were counted and normalized to the number of shCre#4-expressing cells (control; lane 2). The following shRNAs were analyzed: shDPF1-3’UTR#2 and #4 (lanes 3 and 4), shDPF2-3’UTR#3, #4, and #6 (lanes 5, 6, and 7), shDPF3a-CDS#2 (lane 8), shDPF3a-3’UTR#4 (lane 9), shDPF3b-CDS#6 and #7 (lanes 10 and 11), shBrm#4 and #8 (lanes 12 and 13), and shBRG1-CDS#2 and #4 (lanes 14 and 15). The data represent means ± SD (n = 3). Similar results were obtained from at least three independent experiments.

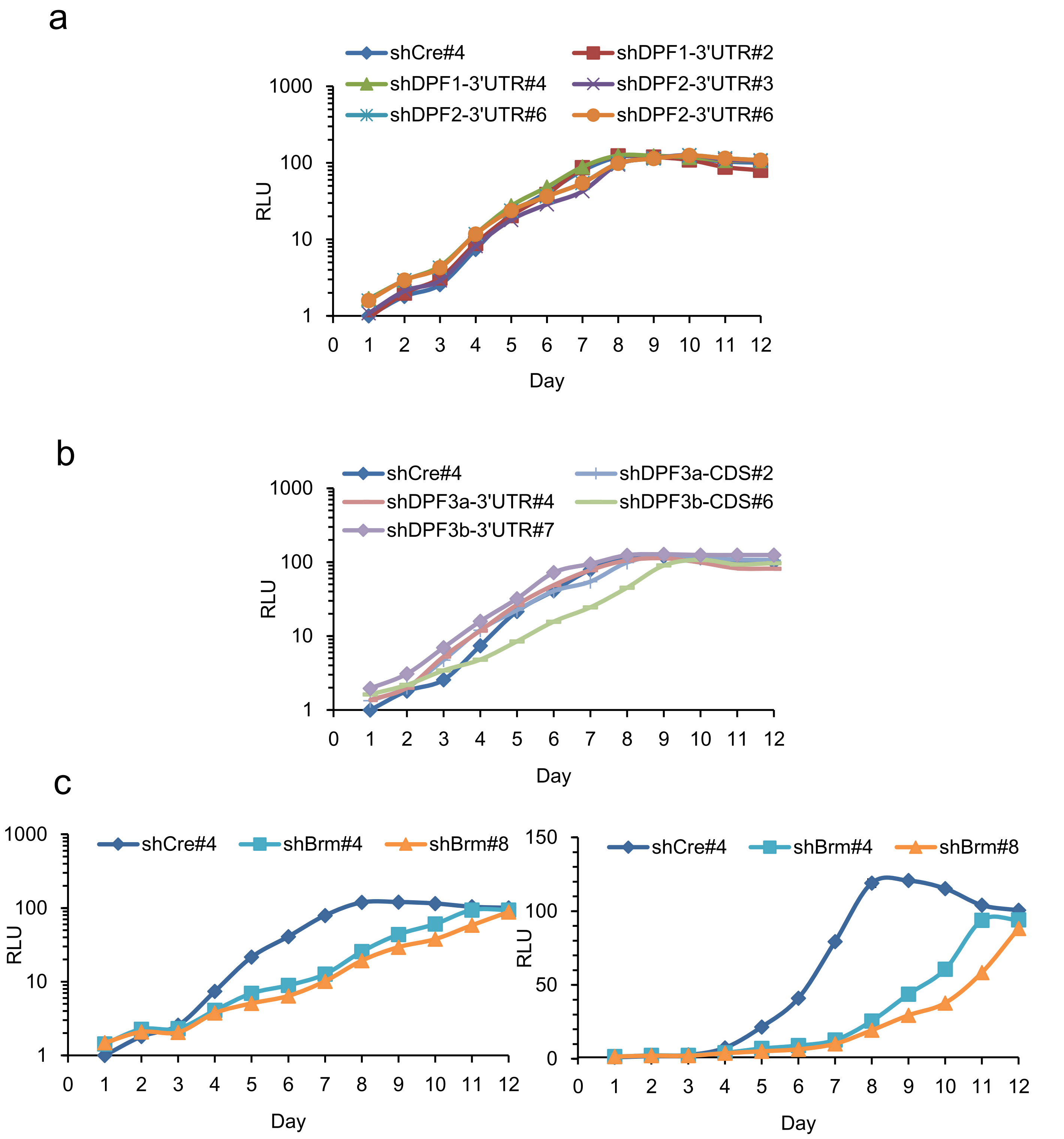

Supplementary Figure S2: Effects of shRNAs on the growth of monolayer cultures of A549.
A549 cells were transduced with shRNA expressing vectors targeting DPF1 and DPF2 (a), DPF3a and DPF3b (b), and Brm (c; left: Semi-log plot, right: normal plot). Cellular growth were monitored as described in Figure 1d.

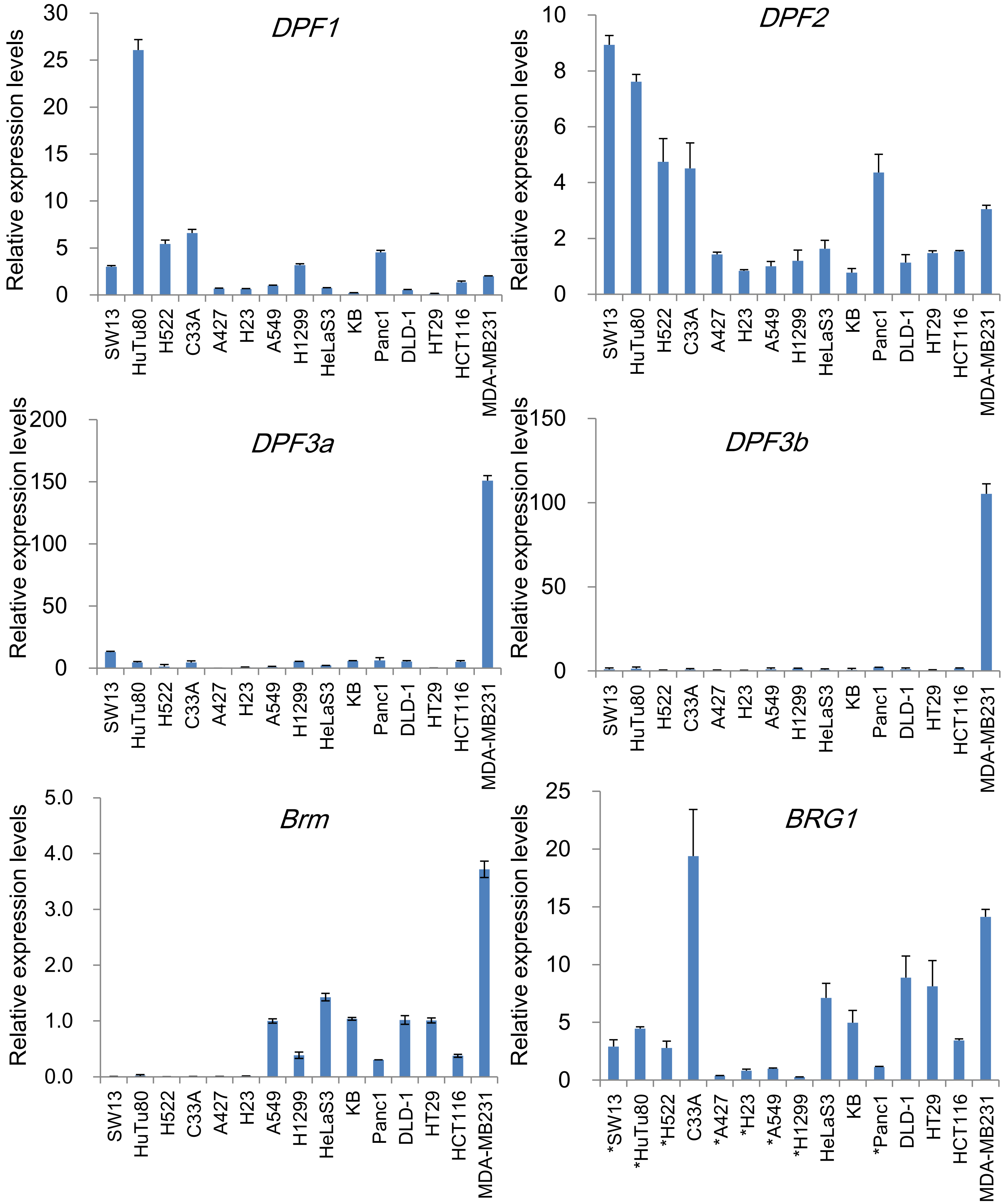

Supplementary Figure S3: Analysis of the d4-family genes, *Brm* and *BRG1* in a panel of epithelial tumor cell lines.

mRNA levels were evaluated by qRT-PCR and data were normalized by assigning a value of 1.0 to the levels in A549 cells. The data represent means ± SD (n = 3). Cell lines the *BRG1* gene of which has nonsense or frame shift mutation, are marked with asterisks. No functional BRG1 protein is detectable in them.

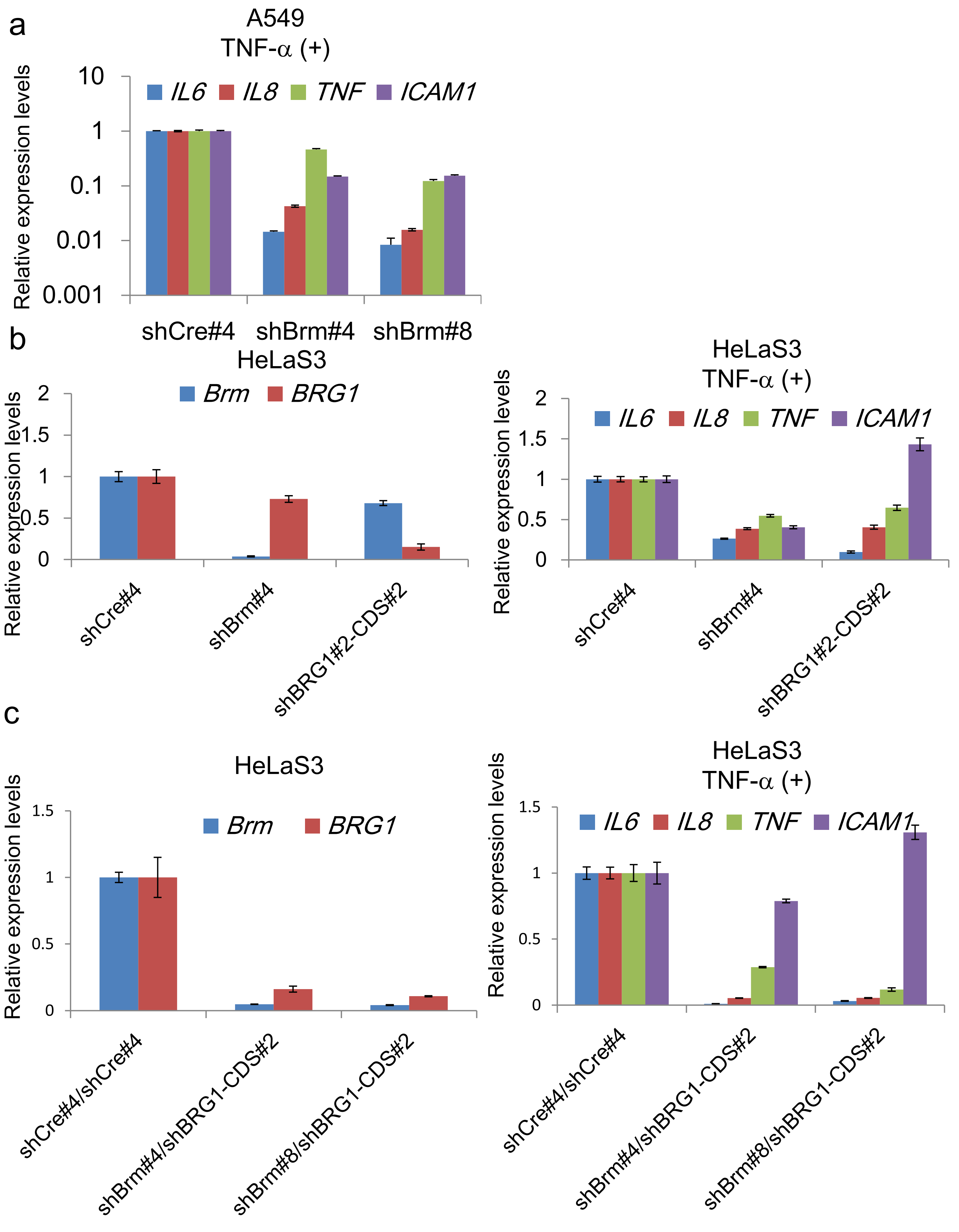

Supplementary Figure S4:

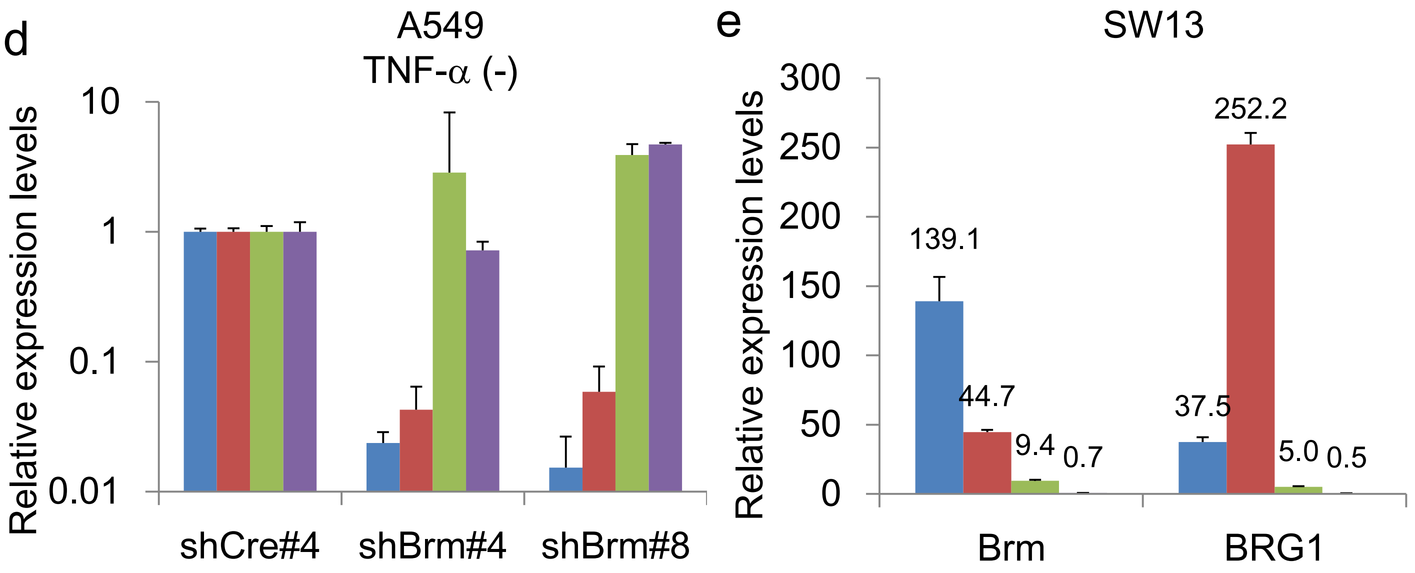

Supplementary Figure S4: Analyses of effects of Brm or BRG1 on the expression of the NF-κB target genes *IL6*, *IL8*, *ICAM1*, and *TNF* by qRT-PCR.

A549 cells (a) or HeLaS3 (b, c) cells transduced with lentivirus vectors expressing either shBrm or shBRG1 (a, b) or simultaneously expressing shBrm and shBRG1 (c) were grown in the presence of TNF-α. Data were normalized by assigning a value of 1.0 to the levels in cells transduced with shCre#4 (control). (d) Parallel cultures of (a) were kept in the absence of TNF-α and analyzed similarly. (e) SW13 cells were transduced with lentivirus vectors expressing Brm or BRG1. Data were normalized by assigning a value of 1.0 to the levels in SW13 cells transduced with control vector. a-e, The data represent means ± SD (n = 3).

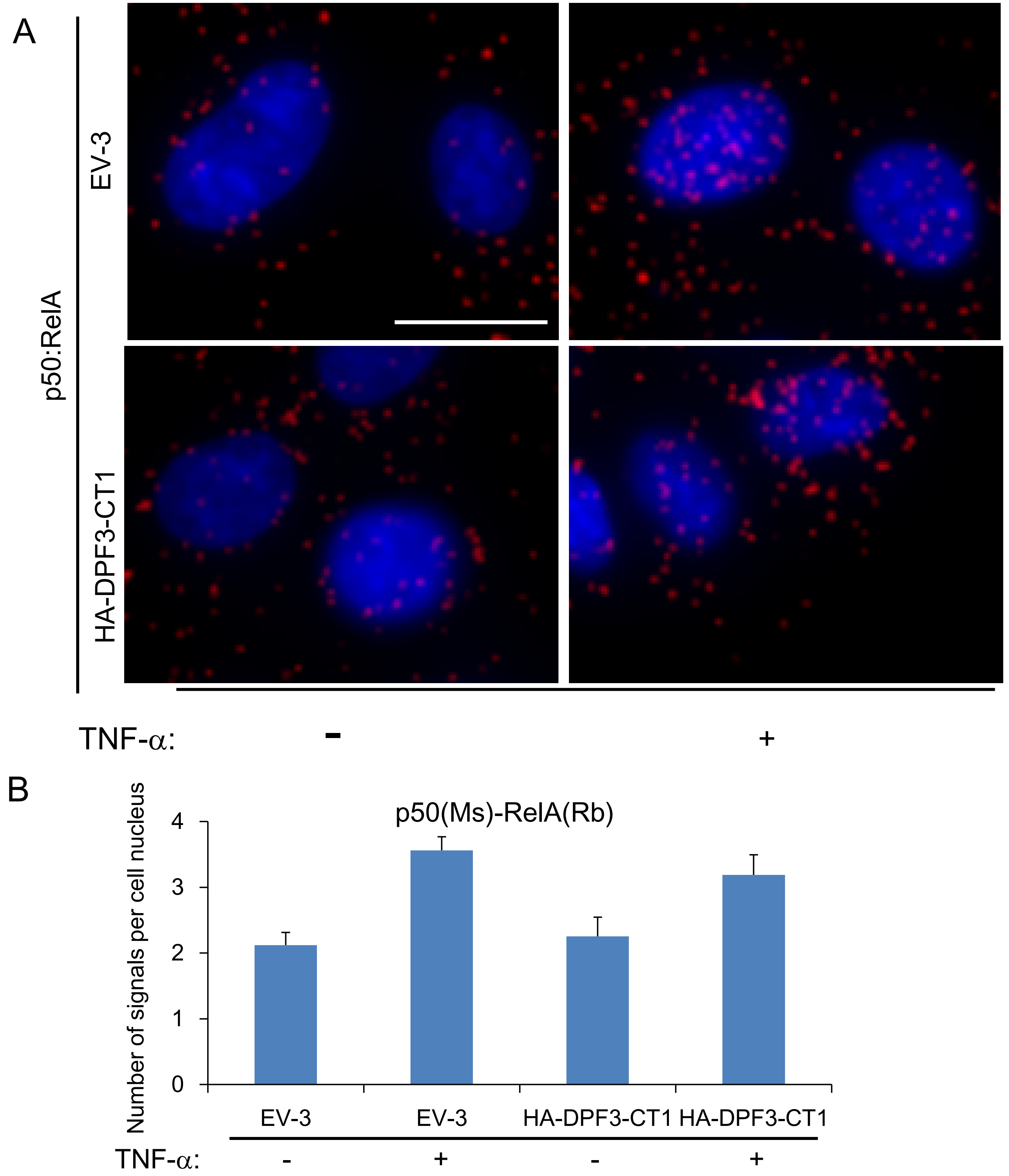

Supplementary Figure S5: Detection of interaction between p50 and RelA in cellular nuclei by *in situ* PLA. (a, b) A549 cells transduced with EV-3 or HA-DPF3-CT1 were grown in the absence (TNF-α: -) or presence of TNF-α for 20 min (TNF-α: +) and then fixed. *In situ* PLA was performed using proximity probes against p50 and RelA antibodies to detect interaction between them. The cells were counterstained with DAPI (nuclei). Bar, 20 mm. (b) Red dot *in situ* hybridization signals in cellular nuclei were counted using 50 cells each. Error bars represent the means ± SEM. Similar results were obtained from at least two independent experiments.

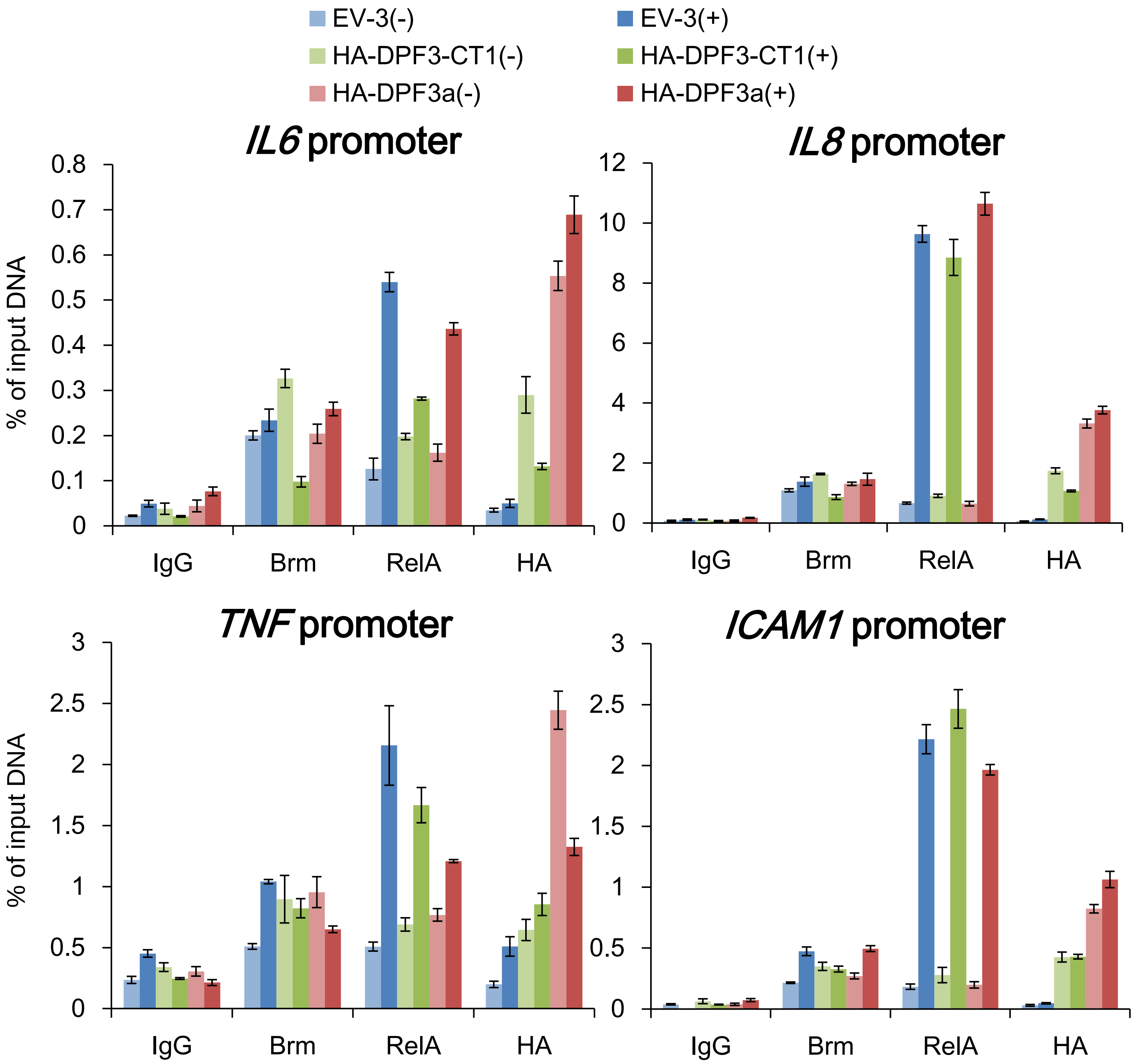

Supplementary Figure S6: ChIP analysis in promoters of four NF-κB target genes.
A549 cells transduced with EV-3, HA-DPF3-CT1, or HA-DPF3a were grown in the absence (TNF-α: -) or presence of TNF-α for 30 min (TNF-α: +) and then fixed. Immunoprecipitation in ChIP was performed using normal IgG (Negative Control) , anti-Brm, anti-RelA, or anti-HA antibodies. Error bars represent the means ± SD. Similar results were obtained when another antibody to RelA or Brm was used instead.
